# Integrated Strategy From *In Vitro*, *In Situ*, *In Vivo* to *In Silico* for Predicting Active Constituents and Exploring Molecular Mechanisms of Tongfengding Capsule for Treating Gout by Inhibiting Inflammatory Responses

**DOI:** 10.3389/fphar.2021.759157

**Published:** 2021-11-29

**Authors:** Wenning Yang, Xiaoquan Jiang, Jingtong Liu, Dongying Qi, Zhiqiang Luo, Guohua Yu, Xueyan Li, Muli Sen, Hongjiao Chen, Wei Liu, Yang Liu, Guopeng Wang

**Affiliations:** ^1^ School of Chinese Materia Medica, Beijing University of Chinese Medicine, Beijing, China; ^2^ School of Life Sciences, Beijing University of Chinese Medicine, Beijing, China; ^3^ Zhongcai Health (Beijing) Biological Technology Development Co., Ltd., Beijing, China

**Keywords:** Tongfengding capsule, UPLC-Q Exactive-Orbitrap HRMS, absorbed components, target network pharmacology, intestinal perfusion with venous sampling

## Abstract

The study of screening active constituents from traditional Chinese medicine (TCM) is important for explicating the mechanism of action of TCM and further evaluating the safety and efficacy effectively. However, detecting and identifying the active constituents from complicated biological samples still remain a challenge. Here, a practical, quick, and novel integrated strategy from *in vitro*, *in situ*, *in vivo* to *in silico* for rapidly screening the active constituents was developed. Firstly, the chemical profile of TCM *in vitro* was identified using UPLC-Q Exactive-Orbitrap HRMS. Secondly, the *in situ* intestinal perfusion with venous sampling (IPVS) method was used to investigate the intestinal absorption components. Thirdly, after intragastric administration of the TCM extract, the *in vivo* absorbed prototype components were detected and identified. Finally, the target network pharmacology approach was applied to explore the potential targets and possible mechanisms of the absorbed components from TCM. The reliability and availability of this approach was demonstrated using Tongfengding capsule (TFDC) as an example of herbal medicine. A total of 141 compounds were detected and identified in TFDC, and among them, 64 components were absorbed into the plasma. Then, a total of 35 absorbed bioactive components and 50 related targets shared commonly by compounds and gout were integrated *via* target network pharmacology analysis. Ultimately, the effects of the absorbed components on metabolism pathways were verified by experiments. These results demonstrated that this original method may provide a practical tool for screening bioactive compounds from TCM treating particular diseases. Furthermore, it also can clarify the potential mechanism of action of TCM and rationalize the application of TFDC as an effective herbal therapy for gout.

## 1 Introduction

Traditional Chinese medicine (TCM) has been widely used for preventing and treating various diseases in China for centuries, which usually exerts a holistic therapeutic effect with the synergistic or antagonistic interactions of multiple characteristic constituents (Guo et al., 2019). However, due to the extremely complicated chemical components in TCM, how to quickly distinguish the components related to its efficacy and to further explicate its mechanism of action have become a problem that need to be urgently solved (Liao et al., 2018). Recently, research has shown that only a few compounds, which have appropriate physicochemical properties and sufficient content in TCM, can be absorbed into the plasma, then transported to the target organs to exert their biological activities (Tian et al., 2018). Thus, compared with considering all components, only focusing on the absorbed components *in vivo* can improve the hit rate of the active compounds effectively and simplify the illustration of the effective material basis (Li et al., 2021). However, in the above research, the circulating plasma samples after oral administration of TCM were collected and used for screening the components absorbed into the plasma. In fact, after taken orally, before entering systemic circulation, the chemical components in TCM are mainly absorbed from the small intestine, so mesenteric vein blood is the first site after gut absorption (Tian et al., 2018), which contains multiple absorbed components. Nowadays, many *in situ* models have been developed and widely used to study drug intestinal absorption, such as the *in situ* closed-loop method (Luo et al., 2016), intestinal single-pass perfusion, intestinal recirculating perfusion, and the intestinal perfusion with venous sampling (IPVS) method (Li et al., 2014). Among them, the IPVS method allows the direct determination of drug absorption through the enterocytes, which has been widely used and recommended due to its similarity to *in vivo* conditions (Caldeira et al., 2018). Moreover, it is often challenging to profile the absorbed components of TCM *in vivo* due to the extremely low concentrations of the absorbed constituents and the interference of endogenous metabolites and proteins (Han et al., 2016). Owing to its high sensitivity and selectivity, ultrahigh-performance liquid chromatography coupled with Q Exactive Hybrid Quadrupole-Orbitrap high-resolution mass spectrometry (UPLC-Q Exactive-Orbitrap HRMS), has become a powerful tool for quickly and precisely profiling the trace compounds in biological samples (Liu et al., 2020; Luo et al., 2021a).

Unlike most “one target, one drug” Western medicines, TCM is a sophisticated system with multicomponent and multi-target characteristics, which achieves its therapeutic effectiveness by regulating multiple physiological pathways (Guo et al., 2019). Therefore, using traditional pharmacological methods to experimentally uncover the unique mechanism of action may not be applicable to TCM research. With the continuous development of bioinformatics, the newly emerging network pharmacology approach has become a powerful tool to elucidate the mechanism of action of complicated drug systems from the molecular level to the pathway level (Chen et al., 2016). However, conventional network pharmacology studies gather compounds from various databases to generate compound–target maps, including some substances with low bioavailability or low content in TCM, which herein produce false-positive results (Xu et al., 2017). Thus, using the absorbed constituents as the target compounds, a new method was proposed, namely, target network pharmacology (Xu et al., 2017), which has been successfully applied to investigate the synergistic mechanisms of multiple components in TCM in the treatment of particular diseases, such as the therapeutic mechanism of ginseng (*Panax ginseng*) for treating Alzheimer’s disease (Feng et al., 2019) and the mechanism of action of Wu-tou decoction on rheumatoid arthritis (Cheng et al., 2021).

Tongfengding capsule (TFDC), a famous prescription approved by the Chinese Food and Drug Administration and included in Chinese Pharmacopoeia Commission (2020), contains eight Chinese herbs: *Gentianae Macrophyllae* Radix (GM), *Phellodendri Chinensis* Cortex (PC), *Corydalis* Rhizoma (CR), *Paeoniae* Radix Rubra (PR), *Cyathulae* Radix (CTR), *Alismatis* Rhizoma (AR), *Plantaginis* Semen (PS), and *Smilacis Glabrae* Rhizoma (SG). This prescription has been widely used in the treatment of rheumatoid arthritis, gout, and hyperuricemia in China for a long time. Modern pharmacology studies have demonstrated that TFDC can effectively inhibit the production and promote the excretion of uric acid and improve the liver and renal functions of patients with gout (Tang, 2016). However, only a few research studies have been reported to analyze its major components using the HPLC technique (Qin et al., 2021), and there has been no systematic characterization of the chemical constituents of this prescription. Moreover, up to now, there have been no reports concerning the absorbed effective substances of TFDC, which play crucial roles in interpreting the pharmacological effects of TFDC in the treatment of various diseases *in vivo*.

**FIGURE 1:**
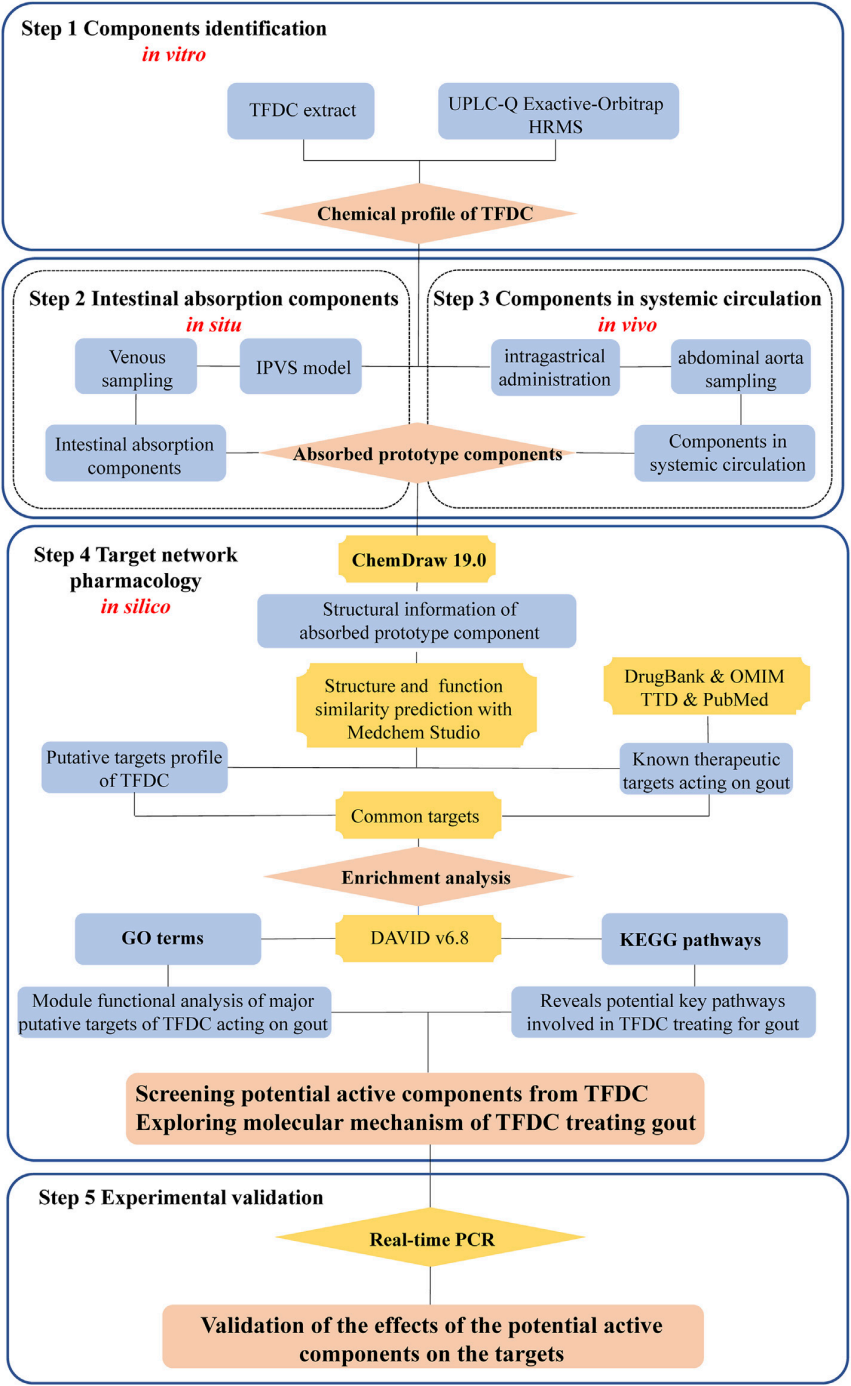
Flowchart of the study design.

To address the above issues, an integrated strategy from *in vitro*, *in situ*, *in vivo* to *in silico* for rapidly screening the active constituents from TCM was developed using TFDC as an example. A detailed description of the strategy is as follows: firstly, the chemical profile of the TFDC extract was established with UPLC-Q Exactive-Orbitrap HRMS *in vitro*. Secondly, an *in situ* IPVS method was used to collect and identify the prototype constituents from intestinal absorption. Thirdly, after intragastric (IG) administration of TFDC, the circulating plasma samples were collected and analyzed to determine the absorbed prototype constituents *in vivo*. Fourthly, *in silico*, a target network pharmacology analysis was used to screen out the bioactive components, corresponding target genes, and the pathways involved in TFDC for treating gout. Finally, some of the predicted results were verified with a lipopolysaccharide (LPS)-induced Tohoku Hospital Pediatrics-1 (THP-1) cell model. We hope that this integrated strategy would be helpful in quickly screening out the bioactive constituents from TCM. The flowchart of the study is presented in Figure 1.

## 2 Materials and Methods

### 2.1 Chemicals and Reagents

The Chinese herbal medicines GM, PC, CR, PR, CTR, AR, PS, and SG were purchased from Beijing Tong Ren Tang Co., Ltd. (Beijing, China), which were identified by Prof. Jingjuan Wang (Beijing University of Chinese Medicine, Beijing, China). TFDC was obtained from Sichuan Sunnyhope Pharmaceutical Co., Ltd. (Sichuan, China). Eleven reference standards—adenine (purity ≥ 99.4%), adenosine (purity ≥ 99.7%), gallic acid (purity ≥ 90.8%), loganic acid (purity ≥ 97.4%), swertiamain (purity ≥ 98.3%), catechin (purity ≥ 99.2%), chlorogenic acid (purity ≥ 96.8%), epicatechin (purity ≥ 99.7%), paeoniflorin (purity ≥ 97.4%), cortisone hydrochloride (purity ≥ 95.1%), and jatrorrhizine hydrochloride (purity ≥ 89.5%)—were purchased from the National Institutes for Food and Drug Control (Beijing, China). Eight reference standards, namely, magnoflorine (purity ≥ 98%), alibiflorin (purity ≥ 91.4%), columbamine (purity ≥ 98%), berberine hydrochloride (purity ≥ 98%), palmatine hydrochloride (purity ≥ 98%), epiberberine (purity ≥ 98%), salicylic acid (purity ≥ 98%), and apigenin (purity ≥ 98%), were obtained from Shanghai Yuanye Pharmaceutical Technology Co., Ltd. (Shanghai, China). Ten reference standards (purity ≥ 98%), namely, phellodendrine, d-tetrahydropalmatine, tetrahydroberberine, corydaline, protopine, cyasterone, dehydrocorydaline, astilbin, engeletin, and limonin, were purchased from Chengdu Purechem-Standard Co., Ltd. (Chengdu, China). Two reference standards, quercetin (purity ≥ 98%) and naringenin (purity ≥ 98.0%), were obtained from Shanghai Standard Technology Co. Ltd. Three more reference standards were used: luteolin (purity ≥ 98%), obtained from Aladdin (Shanghai, China); cryptochlorogenic acid (purity = 99.07%), which was obtained from Chengdu Must Bio-Technology Co., LTD. (Sichuan, China); and oxypaeoniflorin (purity≥98%), which was purchased from Shanghai Tauto Biotech Co., LTD. (Shanghai, China). MS-grade acetonitrile (purity ≥ 99.9%) and formic acid (purity ≥ 99) were supplied by Thermo Fisher Scientific (Fairlawn, NJ, USA). Other chemicals and reagents were analytical grade or higher. The human leukemia monocytic THP-1 cell line was obtained from the Cell Resource Centre, Institute of Basic Medical Sciences, Chinese Academy of Medical Sciences (CAMS)/Peking Union Medical College (PUMC) (Beijing, China). Phorbol myristate acetate (PMA) and LPS were purchased from Sigma-Aldrich (St. Louis, MO, USA).

### 2.2 Preparation of Sample Solutions

#### 2.2.1 Preparation of the Extract Solution of Herbs and TFDC

The eight herbs and TFDC power (0.5 g) were accurately weighed and sonicated with 50% methanol (25 ml) for 30 min at room temperature. The extracted solution was centrifuged at 1,000 × *g* for 10 min at 4°C. Then, the supernatant was transferred to a 50-ml volumetric flask and the precipitate was re-extracted with another 25 ml 50% methanol. After centrifugation, the extracted solution was transferred into the same volumetric flask and filled to the mark. The prepared sample solution was filtered through a 0.22-μm pore-size filter before LC-MS analysis.

#### 2.2.2 Preparation of the Perfusion Solution

The TFDC power (10 g) was accurately weighed and sonicated with water (100 ml) for 30 min at room temperature. The extracted solution was centrifuged at 1,000 × *g* for 10 min at 4°C. Then, the supernatant was transferred into an evaporating dish and the precipitate was re-extracted with another 100 ml water. After centrifugation, the extracted solution was transferred into the same evaporating dish and concentrated using a water bath at 55°C to 100 ml (100 mg/ml).

#### 2.2.3 UPLC-Q Exactive-Orbitrap HRMS Analysis

The compounds were identified using a Vanquish Horizon UPLC system (Thermo Fisher Scientific, Waltham, MA, USA) connected to a Q Exactive Hybrid Quadrupole-Orbitrap high-resolution mass spectrometer (Thermo Fisher Scientific). The chromatographic separation was accomplished by a Waters ACQUITY UPLC BEH Shield RP C18 column (100 mm × 2.1 mm, 1.7 µm) with a column temperature of 35°C. Gradient elution of the analytes was carried out with 0.1% formic acid in water (A) and acetonitrile (B) at a flow rate of 0.3 ml/min. A linear gradient of solvent B (*v*/*v*) was applied as follows: 0–1 min, 5% B; 1–2.4 min, 5–10% B; 2.4–13.5 min, 10–32% B; 13.5–18.5 min, 32–90% B; 18.5–19 min, 90–5% B; and 19–21 min, 5% B. The injection volume was 2 µl.

The heated electrospray ionization (H-ESI) source was operated and the MS parameters were optimized as follows: spray voltage of 3.8 and −3.2 kV in positive and negative modes, respectively; sheath gas flow, 35 a.u.; aux gas flow, 15 a.u.; aux gas heater temperature, 300°C; and capillary temperature, 350°C. A full MS scan/data-dependent MS^2^ (full MS/dd MS^2^) method was used for analysis. The full mass scan range was set from 100 to 1,500 in positive and negative ion modes at resolutions of 70,000 and 17,500 for the MS^2^ scan, respectively. The tandem mass spectrometry (MS/MS) spectra were fragmented by high-energy collision-induced dissociation (HCD) of the normalized collision energy (NCE) values at the levels of 20%, 40%, and 60%. Data acquisition and processing were accomplished with the Xcalibur software (version 4.2; Thermo Fisher Scientific).

### 2.3 Animal Experiments

Normal male Sprague–Dawley rats weighing 220–250 g were purchased from SBF (Beijing) Biotechnology Co., Ltd. (Beijing, China). The animals were housed and handled according to the Laboratory Animal Medicine Guidelines of Beijing University of Chinese Medicine (BUCM). Animals were kept under artificial light on a 12-h light/dark cycle and housed in rooms controlled between 25–27°C and 50%–70% relative humidity. Rats were acclimated for at least 7 days with free access to animal chow and water before the study.

#### 2.3.1 *In Situ* Intestinal Perfusion With Venous Sampling

The surgical operations for IPVS were performed as reported in the literature (Li et al., 2014). Briefly, prior to the initiation of perfusion surgical operation, about 50 ml blood was withdrawn from several rats *via* the abdominal aorta, and the blood incubated in a 37°C water bath was prepared to be transfused into the recipient rat. The recipient was anesthetized with chloral hydrate (400 mg/kg) by intraperitoneal injection and then restrained in a supine position under an infrared lamp to maintain the body temperature. The left jugular vein of the anesthetized rat was exposed and a 24-G BD Intima II catheter filled with heparinized saline (100 U/ml) was inserted into the vein and secured. Then, the catheter was connected by a silicone tube filled with blood to a peristaltic pump, which is placed between the donor blood reservoir and the catheter; the other end of the silicone tube was immersed in the donor blood. The abdominal cavity was carefully opened along the abdominal line to expose the jejunum and the mesenteric vein. Two ends of the jejunum segment were incised with surgical scissors, and then two silicone tubes were inserted through the small slits and secured. After that, the inlet tubing was connected to a syringe pump. Subsequently, the 24-G catheter with heparinized saline was intubated into the mesenteric vein and secured. Then, the catheter was also connected by the silicone tube to the same peristaltic pump utilized for blood supply.

At the conclusion of the surgical operation, the proximal mesenteric vein was ligated with silk suture, and then the two catheters and pumps were switched on immediately. The perfusate was perfused at a flow rate of 0.2 ml/min and the blood was supplied at a flow rate of 0.3 ml/min. During the perfusion, the blood from the mesenteric vein was collected in heparinized centrifuge tubes within 2 h.

#### 2.3.2 IG Administration

A certain amount of TFDC content was dissolved in 0.5% sodium carboxymethyl cellulose (NaCMC) in water to achieve a homogenous suspension at 0.15 g/ml concentration. Male Sprague–Dawley rats were given 4 ml suspensions intragastrically. After 2 h, the rats were anesthetized with chloral hydrate (400 mg/kg) by intraperitoneal injection, and blood was collected *via* the abdominal aorta.

#### 2.3.3 Plasma Sample Preparation

The collected blood was centrifuged at 3,300 × *g* for 10 min, and then the supernatant was precipitated by an equal volume of acetonitrile, followed by centrifugation at 11,800 × *g* for 10 min at 4°C after vortexing for 2 min. Next, the supernatant was evaporated to dryness under a gentle stream of nitrogen at 50°C. Finally, the samples were reconstituted with 200 µl 50% methanol and subjected to LC/MS analysis.

### 2.4 Target Network Pharmacology

#### 2.4.1 Predicting the TFDC-Related Targets

Firstly, the structures of the absorbed prototype components of TFDC were generated using ChemBioDraw Ultra 19.0 and saved in SDF format. Then, based on structure similarity, the targets associated with these TFDC components were predicted with MedChem Studio (MedChem Studio, 3.0, 2012; Simulations Plus, Inc., Lancaster, CA, USA). The similarity threshold was set at 0.7.

#### 2.4.2 Known Therapeutic Targets of Gout

Gout-associated targets were obtained from the following databases using “gout” and “gouty arthritis” as the keywords and limiting the species with “*Homo sapiens*”: DrugBank (https://go.drugbank.com/) (Wishart et al., 2018), the Online Mendelian Inheritance in Man (OMIM; https://www.omim.org) (Amberger et al., 2019), Therapeutic Targets Database (TTD; http://db.idrblab.net/ttd/) (Wang et al., 2020), and PubMed (https://pubmed.ncbi.nlm.nih.gov/). Then, the collected target gene information was standardized using UniProt (https://www.uniprot.org/) (UniProt Consortium, 2021), and the genes without the human sample, Uniprot ID, were excluded.

#### 2.4.3 Gene Ontology and KEGG Enrichment Analysis

The intersection of the predicted targets from MedChem Studio and the known targets of gout were selected for the following bioinformatics analyses. These overlapping targets were further checked and retrieved into UniProt ID by using UniProt. These selected targets were imported into DAVID bioinformatics resources (https://david.ncifcrf.gov/) (Jiao et al., 2012) to perform Gene Ontology (GO) enrichment analysis and Kyoto Encyclopedia of Genes and Genomes (KEGG) pathway enrichment analysis, where targets were only restricted to *H. sapiens*.

#### 2.4.4 Component–Target–Pathway Network Construction

The absorbed prototype components of TFDC and the selected gout-related overlapping targets were correspondingly prepared as XLSX. These targets and the enriched KEGG pathways were also correspondingly arranged as XLSX. The two files were imported into Cytoscape 3.8.2 and merged to construct the component–target–pathway network of TFDC for treating gout.

### 2.5 Cell Culture

THP-1 cells were cultured with RPMI-1640 containing 10% fetal bovine serum (FBS) in a humidified incubator with 5% CO_2_ atmosphere at 37°C and stimulated to macrophages with 200 ng/ml PMA for 24 h.

### 2.6 Cell Viability Assay

The effects of apigenin on cell viability were evaluated by the Cell Counting Kit-8 (CCK-8) assay (Beyotime Institute of Biotechnology, Jiangsu, China). PMA-differentiated THP-1 cells were plated into 96-well plates and incubated with various concentrations of apigenin (0, 10, 20, and 30 μM) for 24 h. Then, CCK-8 was added to the cell cultures for 2 h, and the absorbance of the solution was measured at 450 nm using a microplate reader (SpectraMax iD5; Molecular Devices, San Jose, CA, USA).

### 2.7 Real-Time PCR Assay

To study the effects of apigenin on the messenger RNA (mRNA) expressions of prostaglandin G/H synthase 2 (PTGS2), tumor necrosis factor alpha (TNF-α), and interleukin-6 (IL-6), THP-1 cells were cultured into six-well plates and differentiated for 24 h in the presence of 100 ng/ml PMA. Then, the medium was replaced and the cells were cultured for another 18 h with fresh medium containing different concentrations of apigenin (0, 10, and 20 μM) and LPS (1 μg/ml). After 18 h of incubation with apigenin and LPS, the cells were washed with phosphate-buffered saline (PBS). Total RNA was extracted from the cells using the RNeasy Mini Kit (Qiagen GmbH, Hilden, Germany) according to the manufacturer’s instructions. Then, reverse transcription was performed using the Evo M-MLV RT kit with gDNA Clean for qPCR II (Accurate Biology, Hunan, China) to obtain complementary DNA. Real-time PCR was performed on a BIO-RAD CFX96 Real-Time PCR System using SYBR Green PCR Master Mix. Human-specific primers were synthesized by Sangon Biotech (Shanghai, China). Detailed information is presented in Table 1. The amplification parameters for PCR were listed as follows: 95°C for 10 min and 40 cycles of amplification at 95°C for 15 s and 60°C for 1 min. All experiments were performed in triplicate.

**TABLE 1:** Primers used in real-time PCR.

Gene	Primers	Amplicon size (bp)
GAPDH	Forward: 5′-CAA​ATT​CCA​TGG​CAC​CGT​CA-3′	132
	Reverse: 5′-GAC​TCC​ACG​ACG​TAC​TCA​GC-3′	
PTGS2	Forward: 5′-CTG​GCG​CTC​AGC​CAT​ACA​G-3′	94
	Reverse: 5′-CGC​ACT​TAT​ACT​GGT​CAA​ATC​CC-3′	
TNF-α	Forward: 5′-CCT​CTC​TCT​AAT​CAG​CCC​TCT​G-3′	220
	Reverse: 5′-GAG​GAC​CTG​GGA​GTA​GAT​GAG-3′	
IL-6	Forward: 5′-AGC​CAC​TCA​CCT​CTT​CAG​AAC-3′	118
	Reverse: 5′-GCC​TCT​TTG​CTG​CTT​TCA​CAC-3′	

### 2.8 Statistical Analysis

Data from the real-time PCR experiments were analyzed with SPSS software. Significant differences were assessed with ANOVA. A *p* < 0.05 was considered significant.

## 3 Results and Discussion

### 3.1 Identification of the Compounds in TFDC by UPLC-Q Exactive-Orbitrap HRMS

**FIGURE 2:**
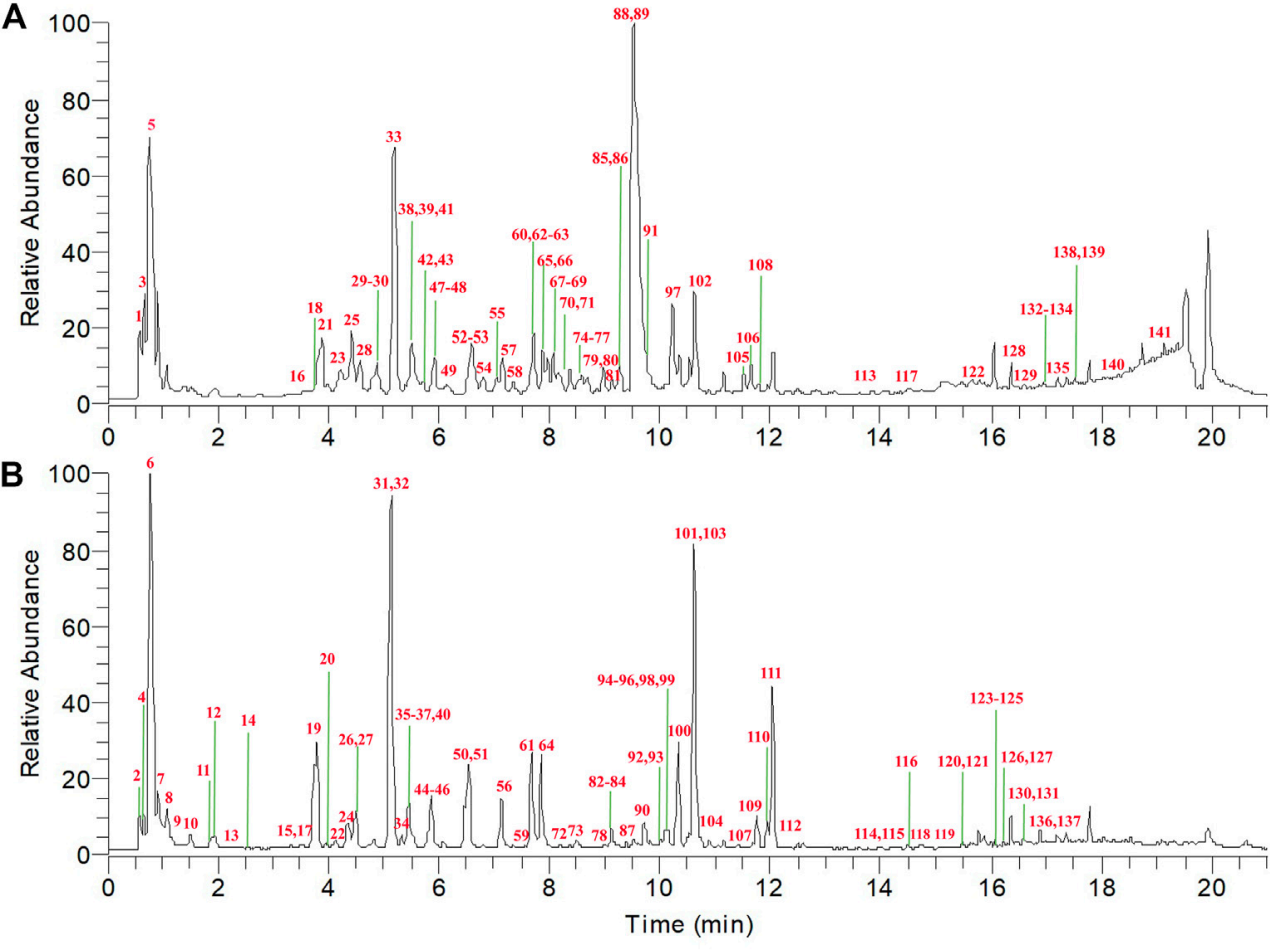
Total ion chromatogram (TIC) of Tongfengding capsule (TFDC) in positive ion mode **(A)** and negative ion mode **(B)** by ultrahigh-performance liquid chromatography coupled with Q Exactive Hybrid Quadrupole-Orbitrap high-resolution mass spectrometry (UPLC-Q Exactive-Orbitrap HRMS).

The chemical profiles of TFDC in both negative and positive modes were well separated and detected by using the established UPLC-Q Exactive-Orbitrap HRMS method. The total ion chromatogram (TIC) in both ESI modes is shown in Figure 2.

In this study, when a pure standard was available, the compound was identified by comparing the retention time and high-resolution accurate mass with those of the standard. Meanwhile, the fragmentation patterns and pathways of the standards helped to further confirm the structures of the derivatives with the same basic skeleton. For the unavailable standard compounds, the structures were proposed by comparing with previous reports according to the chromatographic behavior, accurate mass, MS/MS data, and fragmentation rules. The mass error for the molecular ions of all compounds identified in this study was within ±5 ppm. To reduce false-positive results, the obtained ingredients of TFDC were further screened and confirmed by comparing the chromatographic retention time and fragmental behavior with those of the eight individual crude drugs. Finally, a total of 141 compounds of TFDC were unequivocally identified or tentatively identified, which included 52 alkaloids, 22 flavonoids, 38 terpenoids, 23 organic acids, and 6 other compounds. Among them were 19 compounds from GM, 49 compounds from PC, 29 compounds from CR, 22 compounds from PR, 10 compounds from CTR, 10 compounds from AR, 13 compounds from PS, and 27 compounds from SG. Detailed information on these compounds is summarized in Supplementary Tables S1 and S2.

#### 3.1.1 Identification of Alkaloids

**FIGURE 3:**
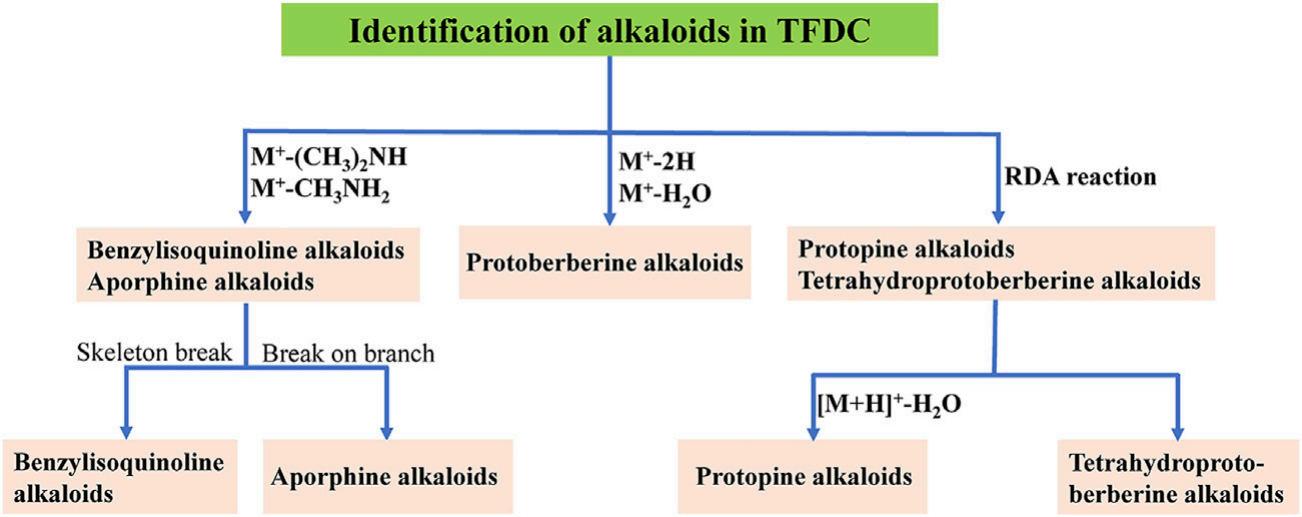
Flowchart for the identification of the structure type of alkaloids in Tongfengding capsule (TFDC).

Alkaloids are a group of basic nitrogen-containing natural products of vegetable origin (Maldoni, 1991). In the present study, the main sources of alkaloids were GM and CR. A total of 52 alkaloids were tentatively identified in the positive model, including 19 protoberberine alkaloids, 14 tetrahydroprotoberberine alkaloids, 3 aporphine alkaloids, 3 protopine alkaloids, 6 benzylisoquinoline alkaloids, and 7 other alkaloids, which are summarized in Supplementary Figure S1. Firstly, we chose berberine (peak 88), tetrahydropalmatine (peak 66), magnoflorine (peak 30), protopine (peak 55), and magnocurarine (peak 16) as the representative compounds to illustrate the characteristic fragmentation rules of protoberberine alkaloids, tetrahydroprotoberberine alkaloids, aporphine alkaloids, protopine alkaloids, and benzylisoquinoline alkaloids, respectively. Their MS^2^ spectra and proposed fragmentation pathways in positive mode are presented in Supplementary Figures S2–S6. Then, the flowchart for the identification of the structure type of alkaloids in TFDC is summarized and presented in Figure 3. Furthermore, 45 alkaloids were identified from TFDC through a comparison of their exact molecular mass, MS/MS spectra, and chromatographic behavior with those of literature data (Cheng et al., 2010; Hong et al., 2012; Jeong et al., 2012; Ji et al., 2012; Shan et al., 2018; Tan et al., 2013; Xian et al., 2014; Wang et al., 2016; Sun et al., 2016; Zuo et al., 2018). Detailed information on these compounds is summarized in Supplementary Table S1.

Seven other alkaloids were also discovered in TFDC (peaks 1, 3, 5, 41, 47, 113, and 117). Among them, compounds 1 and 3 were identified as adenine and adenosine *via* reference standards, respectively. Based on their exact molecular mass, MS/MS spectra, and chromatographic behavior compared with those of literature data, compounds 5, 41, 47, 113, and 117 were tentatively identified as nicotinic acid (Zou et al., 2015), plantagoguanidinic acid (Wang et al., 2016), dasycarpamin (Wang et al., 2013), skimmianine (Xian et al., 2014), and γ-fagarine (Xian et al., 2014), respectively.

#### 3.1.2 Identification of Flavonoids

The basic structure of flavonoids consists of two benzene rings (A and B) linked by a heterocyclic pyrane ring (C ring). Flavonoids are further classified into several subclasses (flavones, flavonols, flavanones, flavanonol, etc.) (Zou et al., 2015). In the present study, a total of 22 flavonoids were unambiguously or tentatively identified from TFDC in the positive and negative modes, and the main source of flavonoids was SG. In order to explore the fragmentation pathways of flavonoids, seven standards—catechin (peak 40), epicatechin (peak 50), astilbin (peak 103), quercetin (peak 108), engeletin (peak 111), luteolin (peak 121), and naringenin (peak 124)—were first identified. Briefly, the MS/MS behavior of flavone aglycones was characterized by the retro Diels–Alder (RDA) fragmentation pathway and successive loss of small molecules and/or radicals such as CH_3_, CO, and CO_2_ (Wang et al., 2014). For flavonoid *O*-glycosides, the MS characteristic fragment ions were featured by producing the neutral loss of 146 and 162 Da (Luo et al., 2021b). Taking compound 103 as an example, a (M–H)^−^ ion at *m*/*z* 449.1082 (C_21_H_21_O_11_) was shown, and the main fragment ions were observed at *m*/*z* 303.0501 (M–H–C_6_H_10_O_4_)^−^, *m*/*z* 285.0396 (M–H–C_6_H_10_O_4_–H_2_O)^−^, *m*/*z* 178.9973 (M–H–C_13_H_18_O_6_)^−^, *m*/*z* 151.0023 (M–H–C_6_H_10_O_4_–C_8_H_8_O_3_)^−^, and *m*/*z* 125.0229 (M–H–C_15_H_18_O_9_)^−^ in the negative ion spectrum. Thus, compound 103 was definitely identified as astilbin. Its MS^2^ spectrum and proposed fragmentation pathways in negative mode are shown in Supplementary Figure S7.

Compounds 109 and 111 (engeletin) were considered to be isomers as they displayed the same (M–H)^−^ ions at *m*/*z* 433.1133 (C_21_H_21_O_10_) and *m*/*z* 433.1132 (C_21_H_21_O_10_). Moreover, they had similar fragment ions at *m*/*z* 287.05 (M–H–C_6_H_10_O_4_)^−^, *m*/*z* 269.04 (M–H–C_6_H_12_O_5_)^−^, *m*/*z* 259.06 (M–H–C_7_H_10_O_5_)^−^, *m*/*z* 178.99 (M–H–C_13_H_18_O_5_)^−^, and *m*/*z* 152.01 (M–H–C_14_H_17_O_6_)^−^ in the MS^2^ spectra. By comparing their chromatographic retention times with those of literature data (Chen et al., 2014), compound 109 was tentatively identified as neoengeletin. Similarly, *via* a comparison of their chromatographic behavior, exact molecular masses, and MS/MS fragmentation patterns, as shown above, with those of literature data (Chen et al., 2014; Dai et al., 2015; Zou et al., 2015; Wang et al., 2016), a total of 22 flavonoids were identified, which are summarized in Supplementary Figure S8. Detailed information on these compounds is summarized in Supplementary Tables S1 and S2.

#### 3.1.3 Identification of Terpenoids

In the present study, a total of 38 terpenoids were found, including 13 iridoids, 8 monoterpene glycosides, and 17 triterpenoids and derivatives. The classification of these compounds is shown in Supplementary Figure S9. The source of iridoids was GM. To explore the fragmentation rules of iridoids, three standards, namely, loganic acid (peak 19), swertiamain (peak 24), and gentiopicrin (peak 32), were first identified. Taking compound 19 as an example, a (M–H)^−^ ion at an *m*/*z* of 375.1289 (C_16_H_23_O_10_) was exhibited, and in the negative ion spectrum, the main fragment ions were observed at *m*/*z* values of 213.0762 (M–H–Glc)^−^, 169.0860 (M–H–Glc–CO_2_]^−^, and 151.0755 (M–H–Glc–CO_2_–H_2_O)^−^. Thus, compound 19 was unambiguously identified as loganic acid. Its MS^2^ spectrum and proposed fragmentation pathways in negative mode are shown in Supplementary Figure S10. Similarly, based on the chromatographic behavior and similar fragmentation pathways, 10 other iridoids (peaks 13, 14, 17, 27, 28, 33, 34, 38, 119, and 120) were tentatively identified as isoboonein, geniposidic acid, secologanic acid, secologanoside, 6′-*O*-β-d-glucosyl gentiopicroside, 4′-*O*-β-d-glucosyl-gentiopicroside, sweroside, olivieroside C, macrophylloside B, and macrophylloside A (Lv et al., 2012; Wang et al., 2016; Pan et al., 2016; Zhang et al., 2020), respectively.

Eight monoterpene glycosides were detected in this work, and their source was PR. Firstly, three standards—oxypaeoniflorin (peak 31), alibiflorin (peak 45), and paeoniflorin (peak 51)—were first identified by comparing with the references to illustrate the fragmentation rules. Compound 31 (oxypaeoniflorin) afforded a quasi-molecular ion at *m*/*z* 495.1507 (C_23_H_27_O_12_) in the negative model, and its MS^2^ spectra showed representative ions at *m*/*z* 465.1407 (M–H–CH_2_O)^−^, *m*/*z* 137.0232 (M–H–C_16_H_22_O_9_)^−^, and *m*/*z* 93.0332 (M–H–C_16_H_22_O_9_–CO_2_)^−^. The MS^2^ mass spectra and the fragmentation pathways of oxypaeoniflorin are shown in Supplementary Figure S11. Consequently, *via* comparison of their chromatographic behavior, exact molecular masses, and MS/MS fragmentation patterns, as shown above, with those of the literature data (Li et al., 2009), the other five monoterpene glycosides (peaks 9, 83, 99, 116, and 118) were also tentatively identified as 1-*O*-β-d-glucopyranosyl-paeonisuffrone, galloylalbiflorin, galloylpaeoniflorin, benzoylpaeoniflorin, and isobenzoylpaeoniflorin, respectively.

Seventeen triterpenoids and derivatives, including 13 triterpenoids and 4 triterpenoid saponins, were detected in TFDC. Firstly, compound 128 was identified as limonin by comparing with the standard. Consequently, compound 122, taken as an example, afforded a quasi-molecular ion at *m*/*z* 505.3524 (C_30_H_49_O_6_) in the positive model, and its MS^2^ spectra showed representative ions at *m*/*z* 487.3419 (M+H–H_2_O)^+^, *m*/*z* 469.3312 (M+H–2H_2_O)^+^, *m*/*z* 451.3202 (M+H–3H_2_O)^+^, *m*/*z* 415.2842 (M+H–C_4_H_10_O_2_)^+^, *m*/*z* 397.2735 (M+H–C_4_H_10_O_2_–H_2_O)^+^, and *m*/*z* 353.2471 (M+H–C_4_H_10_O_2_–H_2_O–C_2_H_4_O)^+^. Thus, compound 122 was tentatively identified as 16-oxoalisol A (Yang et al., 2020). Its MS^2^ mass spectra and the fragmentation pathways are shown in Supplementary Figure S12. Similarly, based on the similar fragmentation pathways, six triterpenoids (peaks 133, 134, 138, 139, 140, and 141) were tentatively identified as 11-anhydro-16-oxoalisol A, 11-deoxy-16-oxoalisol A, alisol C 23-acetate, 24-deacetyl-alisol O or 16,23-oxido-alisol B, 25-dehydroxy alisol A 24-acetate, and alisol B 23-acetate, respectively (Luo et al., 2021a). The remaining compounds (peaks 115, 123, 125, 126, 129, 131, 135, 136, and 137) were tentatively identified *via* comparison of their exact molecular masses and MS/MS spectra with those of literature data (Li et al., 2005; Fan et al., 2010; Gualdani et al., 2016; Wang et al., 2017; Luo et al., 2021b). Detailed information on these compounds is summarized in Supplementary Tables S1 and S2.

#### 3.1.4 Identification of Organic Acids

In the present study, a total of 23 organic acids were detected: 3 aliphatic organic acids, 11 phenolic acid derivatives, and 9 quinic acid and derivatives. The main sources of organic acids were CR and PR, and the classification of these compounds is shown in Supplementary Figure S13. Firstly, by comparing their exact molecular masses and MS/MS spectra with those of literature data, three aliphatic organic acids (peaks 6, 8, and 92) were tentatively identified as malic acid (Zou et al., 2015), citric acid (Zou et al., 2015), and azelaic acid (Rea Martinez et al., 2020), respectively. For the phenolic acid derivatives, the primary MS/MS behavior was the neutral losses of glucoside, H_2_O, and CO_2_. By comparing with the standards, compounds 12 and 101 were unequivocally identified as gallic acid and salicylic acid, respectively. Compounds 7, 10, and 11 showed the same molecular formula and similar (M-H)^−^ ions at *m*/*z* values of 493.1197 (C_19_H_25_O_15_), 493.1201 (C_19_H_25_O_15_), and 493.1198 (C_19_H_25_O_15_), respectively. Moreover, they exhibited similar fragment ions at *m*/*z* 331.06 (M–H–Glc)^−^, *m*/*z* 313.05 (M–H–Glc–H_2_O)^−^, *m*/*z* 169.01 (M–H–C_12_H_20_O_10_)^−^, and *m*/*z* 125.02 (M–H–C_12_H_20_O_10_–CO_2_)^−^. Thus, by comparing their chromatographic retention times, exact molecular masses, and MS/MS spectra with those of literature data (Li et al., 2009) and the Orbitrap Traditional Chinese Medicine Library (OTCML), compounds 7, 10, and 11 were tentatively identified as 1′-*O*-galloylsucrose, 6′-*O*-galloylsucrose, and 6-*O*-galloylsucrose, respectively. Taking compound 7 as an example, its MS^2^ spectrum and proposed fragmentation pathways in negative mode are shown in Supplementary Figure S14. Similarly, based on the chromatographic behavior and the similar fragmentation pathways, five other phenolic acid derivatives (peaks 15, 35, 64, 78, 84, and 96) were tentatively identified as protocatechuic acid, 4-hydroxybenzoic acid, 5-*O*-caffeoylshikimic acid, *p*-hydroxy-cinnamic acid, acteoside, and isoacteoside, respectively (Li et al., 2009; Ccana-Ccapatinta et al., 2020).

The source of the nine quinic acids and derivatives was PC. Firstly, two standards, cryptochlorogenic acid (peak 37) and chlorogenic acid (peak 44), were first identified by comparing with the references to illustrate the fragmentation rules. Consequently, compound 37 (cryptochlorogenic acid), taken as an example, gave a quasi-molecular ion at *m*/*z* 353.0872 (C_16_H_17_O_9_) in the negative model, and its MS^2^ spectra showed representative ions at *m*/*z* 191.0553 (M–H–caffeoyl)^−^, *m*/*z* 179.0340 (M–H–C_7_H_10_O_5_)^−^, *m*/*z* 173.0445 (M–H–C_9_H_8_O_4_)^−^, *m*/*z* 135.0440 (M–H–C_7_H_10_O_5_–CO_2_)^−^, and *m*/*z* 93.0332 (M–H–2H_2_O–CO_2_)^−^. Thus, compound 37 was unequivocally identified as cryptochlorogenic acid. Its MS^2^ spectrum and proposed fragmentation pathways in negative mode are shown in Supplementary Figure S15. Similarly, the remaining compounds (peaks 4, 20, 36, 46, 56, 59, and 61) were tentatively identified *via* comparison of their chromatographic retention times, exact molecular masses, and MS/MS spectra with those of literature data (Sun et al., 2016; Ccana-Ccapatinta et al., 2020). Detailed information on these compounds is summarized in Supplementary Tables S1 and S2.

#### 3.1.5 Identification of Other Compounds

Six other compounds (peaks 2, 22, 26, 72, 87, and 94) were also detected in the negative mode in TFDC. Firstly, compound 94 was identified as cyasterone *via* a reference standard. Consequently, by comparing their exact molecular masses and MS/MS spectra with those of literature data and OTCML, the remaining compounds (peaks 2, 22, 26, 72, and 87) were tentatively identified as sucrose, protocatechualdehyde, syringin, (+/−)8-(4-hydroxy-3-methoxyphenyl)-6,7-bis(hydroxymethyl)-3-methoxy-5,6,7,8-tetrahydro-2-naphthalenyl-β-d-glucopyranoside (Xian et al., 2014), and syringaresinol (Sim et al., 2013), respectively.

### 3.2 Identification of the Absorbed Components

**FIGURE 4:**
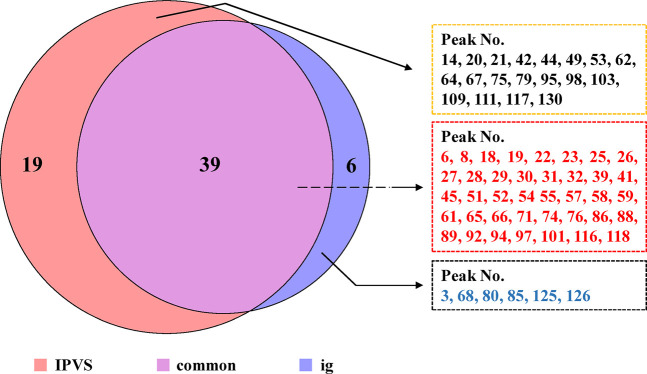
Comparison of the absorbed components between intestinal perfusion with venous sampling (IPVS) and the intragastric (IG) method.

After oral administration, the chemical components of TCM are mainly absorbed from the small intestine, so mesenteric venous blood usually contains higher concentrations of the absorbed components compared to systemic circulation blood. Moreover, compared with a single oral administration, some relatively low-content components of TCM could be detected and identified using the IPVS method with continuous drug perfusion. On the other hand, to comprehensively screen the components *in vivo*, the major compounds in rat plasma (blood was collected from the abdominal aorta) after IG administration of TFDC were analyzed. Finally, the results indicated that a total of 64 prototype components were absorbed into plasma, which might play important roles in exerting the biological and pharmacological effects of TFDC. Among them, 58 were from the IPVS group and 45 of them were from the IG group. Detailed information of all these absorbed components is summarized in Figure 4.

### 3.3 Target Network Pharmacology Analysis

#### 3.3.1 Putative Targets of TFDC

Although 64 absorbed prototype components were detected, five compounds (peak 21, 23, 54, 95, and 126) were excluded, for which structural information of these components was uncertain. Therefore, the remaining 59 compounds were selected to predict the targets of TFDC with MedChem Studio; their detailed structural information is provided in Supplementary Table S3. As a result, a total of 753 putative targets were collected (Supplementary Table S4).

#### 3.3.2 Known Therapeutic Targets Acting on Gout

Based on available data from various gout disease databases, 222 gout-related targets were collected and are presented in Supplementary Table S5. By mapping the compound targets with these gout-related targets, 50 common targets were identified. Among the 59 components used for target prediction, 35 were selected for their predicted targets overlapping the targets of gout. Therefore, 35 components of TFDC and 50 gout-related targets were used for further analysis.

#### 3.3.3 Network and Pathway Analysis

**FIGURE 5:**
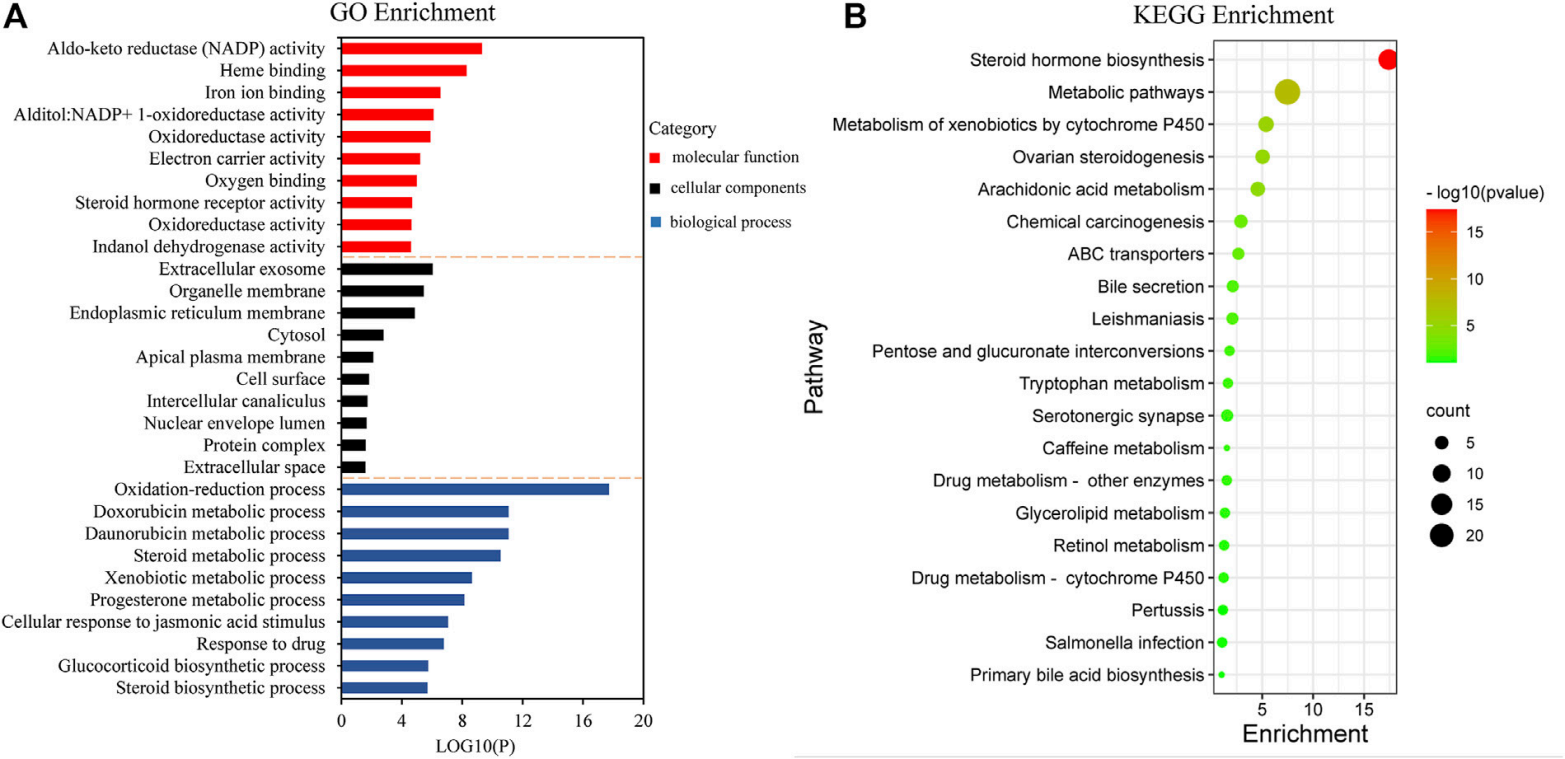
Gene Ontology (GO) term performance and pathway enrichment analysis of common targets. **(A)** GO term performance by biological process (BP), cellular component (CC), and molecular function (MF). **(B)** Pathway enrichment analysis by the Kyoto Encyclopedia of Genes and Genomes (KEGG).

For the GO terms and KEGG pathway analysis, 50 common targets described above, which were entered into the DAVID database and with *p*-values less than 0.05, were enriched. The top 10 terms for biological process (BP), cellular components (CC), and molecular function (MF) are shown as bar plot in Figure 5A. The enriched BP ontologies focused on various metabolic processes, including doxorubicin metabolic process, daunorubicin metabolic process, steroid metabolic process, xenobiotic metabolic process, and progesterone metabolic process. Besides, it is worth noting that the GO terms “leukotriene metabolic process” and “leukotriene biosynthetic process” were also significant entries in BP, which played an important role in leukotriene B_4_ (LTB_4_) metabolism, and recent research has indicated that the amount of LTB_4_ in plasma was closely associated with gout (Luo et al., 2019). The majority of the protein responses were located in a variety of cell components such as the extracellular exosome, organelle membrane, endoplasmic reticulum membrane, cytosol, apical plasma membrane, cell surface, intercellular canaliculus, nuclear envelope lumen, protein complex, and extracellular space. Moreover, the enriched MF ontologies were dominated by aldo-keto reductase (NADP) activity, heme binding, iron ion binding, alditol:NADP^+^ 1-oxidoreductase activity, oxidoreductase activity, electron carrier activity, oxygen binding, steroid hormone receptor activity, oxidoreductase activity, and indanol dehydrogenase activity.

To further explore the significant signaling pathways of the major target genes, KEGG pathway enrichment analysis was performed and resulted in 20 pathways with significant enrichment (*p* < 0.05). The pathways are presented in Figure 5B, which could be categorized into four major functional groups: metabolism (such as steroid hormone biosynthesis, steroid hormone biosynthesis, metabolism of xenobiotics by cytochrome P450, arachidonic acid metabolism, and pentose and glucuronate interconversions), organismal systems (such as ovarian steroidogenesis and bile secretion), human diseases (such as chemical carcinogenesis and leishmaniasis), and membrane transport (such as ABC transporters).

**FIGURE 6:**
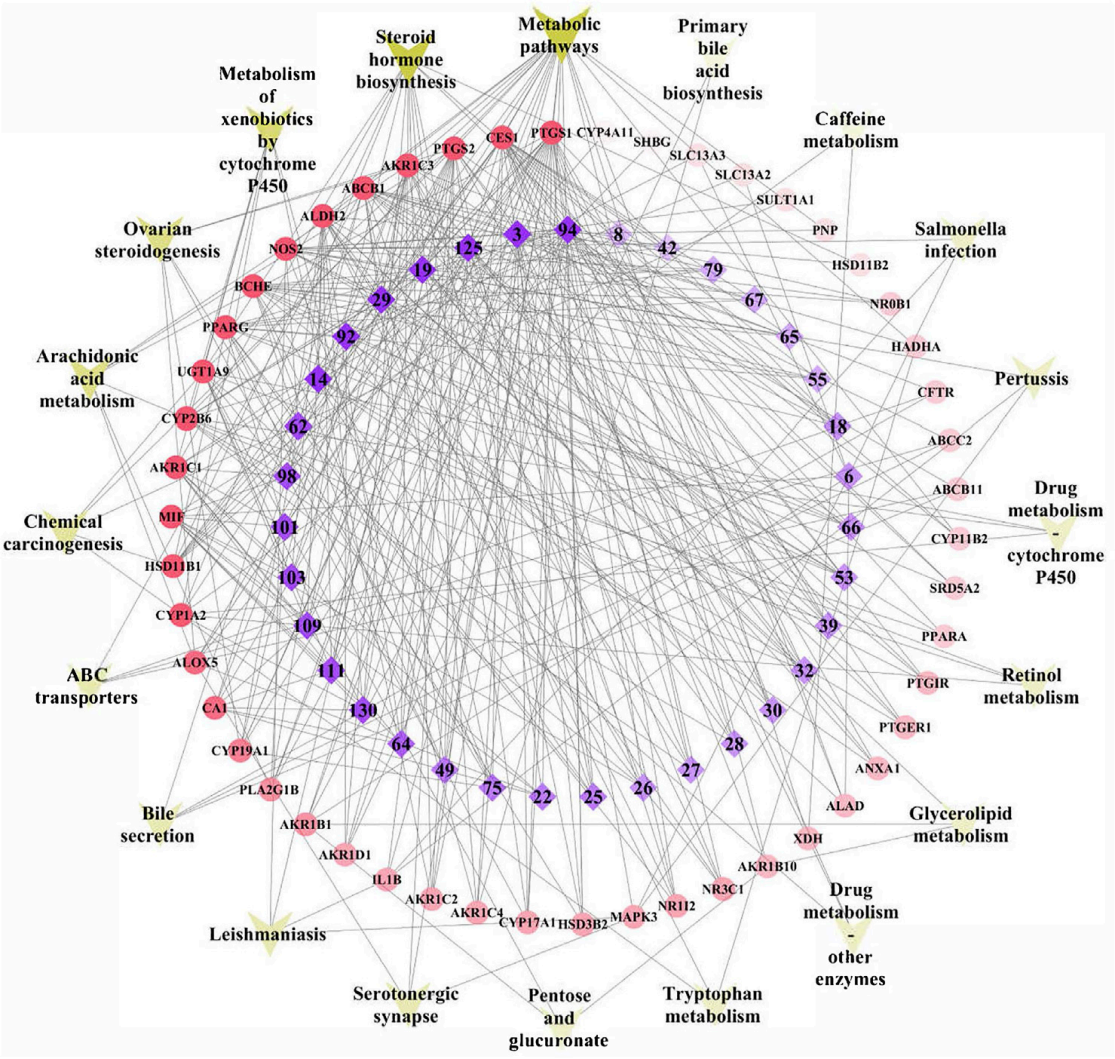
Tongfengding capsule (TFDC) key components–major targets–main pathways network. *Innermost* and *purple diamond* represents the 35 validated compounds of TFDC, the *middle* and *red circle* stands for the 50 gout-related targets, and the *outermost “V” shape* in *yellow* indicates the 20 enriched Kyoto Encyclopedia of Genes and Genomes (KEGG) pathways. *Edges* represent interactions among the key components, targets, and pathways.

Subsequently, a network was constructed by linking the validated components, target genes, and significant pathways to achieve a comprehensive understanding of the mechanism of action, as shown in Figure 6. The network consisted of 105 nodes and 373 edges, with the innermost and purple diamond representing the 35 validated compounds of TFDC, the middle and red circle indicating the 50 gout-related targets, and the outermost “V” shape in yellow indicating the 20 enriched KEGG pathways. It is worth noting that the arachidonic acid metabolism pathway was highly enriched in the KEGG pathway analysis, which played an important role in gout treatment (Graham et al., 2013; Napagoda et al., 2018; Deng et al., 2020).

#### 3.3.4 TFDC Attenuates Gout *via* Regulating the Arachidonic Acid Metabolism Pathway

**FIGURE 7:**
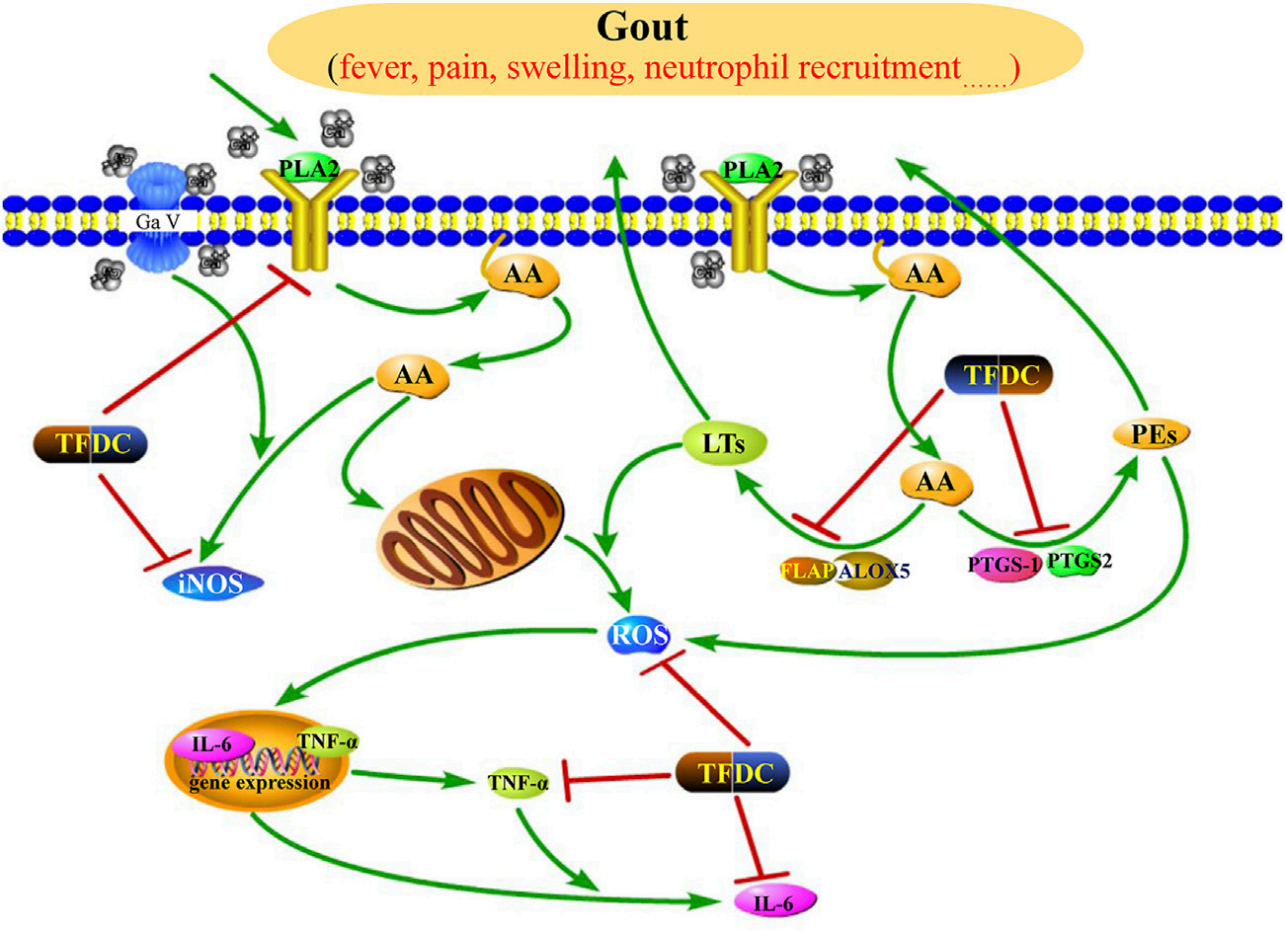
Illustration of the arachidonic acid metabolism pathway influenced by the major putative targets of TFDC components. *PLA2*, phospholipase A2; *AA*, arachidonic acid; *LTs*, leukotrienes; *PGs*, prostaglandins; *iNOS*, inducible NO synthase; *ROS*, reactive oxygen species; *FLAP*, 5-lipoxygenase-activating protein; *ALOX5*, polyunsaturated fatty acid 5-lipoxygenase; *PTGS1*, prostaglandin G/H synthase 1; *PTGS2*, prostaglandin G/H synthase 2; *TNF-α*, tumor necrosis factor alpha; *IL-6*, interleukin-6; *TFDC*, Tongfengding capsule.

Gout, defined as a type of inflammatory arthritis caused by the deposition of monosodium urate (MSU) crystals in the synovial fluid and other tissues (Dalbeth et al., 2019), has four clinical phases: asymptomatic hyperuricemia, acute gouty arthritis, intercritical gout, and chronic tophaceous gout (Bohata et al., 2021). According to the network analysis, the anti-gout mechanism of TFDC may be associated with the regulation of the arachidonic acid pathway. As shown in Supplementary Table S8, the TFDC putative targets involved in the arachidonic acid metabolism pathway included phospholipase A2 (PLA2G1B, also called PLA2), prostaglandin G/H synthase 1 (PTGS1, also called COX 1), PTGS2, polyunsaturated fatty acid 5-lipoxygenase (ALOX5, also called 5-LOX), aldo-keto reductase family 1 member C3 (AKR1C3), and cytochrome P450 2B6 (CYP2B6). Figure 7 shows the arachidonic acid metabolism pathway affected by the major putative targets of TFDC; the details are discussed below.

Arachidonic acid generally is incorporated into the plasma membrane, and the release of arachidonic acid from membrane phospholipids is through the activation of various phospholipase enzymes, mainly PLA2 (Munaron 2011). Therefore, the antagonists of PLA2 can block PLA2 to release arachidonic acid. Based on our predicted results, four compounds (92, 94, 101, and 125) were considered as PLA2 antagonists, so they may partially contribute to the anti-gout effects of TFDC.

Prostaglandin G/H synthase (PTGS, also called COX), including two isomerases, namely, PTGS1 and PTGS2, and ALOX5 are two rate-limiting enzymes involved in the metabolism of arachidonic acid. Metabolism by PTGS leads to the formation of prostaglandins (PGs), whereas metabolism by ALOX5 leads to the generation of leukotrienes (LTs). These two types of metabolites are actively involved in the development of inflammatory diseases. The PGs are connected with vascular permeability, vasodilatation, fever, and pain amplification (Laupattarakasem et al., 2003), and the LTs are key mediators in inflammatory and allergic processes (Patil et al., 2019). Therefore, we can suppress the production of LTs and PGs by inhibiting PTGS1, PTGS2, and ALOX5, leading to anti-inflammatory and analgesic effects in gout. Many of the identified active compounds from TFDC provide strong support for the inhibitory effect of TFDC on arachidonic acid metabolism. It has been shown that compounds such as geniposidic acid (peak 14) (Ramirez-Cisneros et al., 2015), loganic acid (peak 19) (Ramirez-Cisneros et al., 2015), protocatechualdehyde (peak 22) (Chang et al., 2011), phellodendrine (peak 25) (Li et al., 2016), magnoflorine (peak 30) (Li et al., 2019), oblongine (peak 39) (Li et al., 2019), menisperine (peak 42) (Li et al., 2019), cyasterone (peak 94) (Cao et al., 2017), salicylic acid (peak 101) (Paterson and Lawrence, 2001), astilbin (peak 103) (Wang et al., 2018), engeletin (peak 111) (Wu et al., 2016), and apigenin (peak 130) (Lajter et al., 2015) have suppressive effects on PTGS, while compounds such as salicylic acid (peak 101) (Lapenna et al., 2009) and apigenin (peak 130) (Lajter et al., 2015) were reported to downregulate ALOX5. Therefore, TFDC may play an anti-gout role through regulating the arachidonic acid metabolism pathway by inhibiting PTGS and ALOX5.

TNF-α, a cytokine, can induce fever and stimulate synovial cells to produce collagenase and prostaglandin E_2_ (PGE_2_) and thus is considered to contribute to joint damage in inflammatory conditions such as rheumatoid arthritis (Warren, 1990). Recently, a study has shown that arachidonic acid can stimulate TNF-α production in Kupffer cells (Cubero and Nieto 2012). Moreover, TNF-α further exerts secondary inflammatory effects by stimulating IL-6 synthesis in several cell types. Upregulation of IL-6 production has been observed in patients with gout disease (Mitsuyama et al., 2008). Several of the identified active compounds from TFDC have shown potent inhibition on TNF-α and IL-6, such as magnoflorine (peak 30) (Guo et al., 2018), astilbin (peak 103) (Wang et al., 2018), and engeletin (peak 111) (Jiang et al., 2018). Besides, the pharmacological activity of a single herb of TFDC on gout has also been reported. The water extract of SG can reduce the serum levels of TNF-α and IL-6 in hyperuricemia and gouty mice and showed significant effects in ameliorating murine hyperuricemia and gout induced by potassium oxonate and monosodium urate (Liang et al., 2019). Taken together, the anti-gout function of TFDC may be partially attributed to the downregulation of the expressions of TNF-α and IL-6.

Reactive oxygen species (ROS) is an umbrella term including oxygen radicals and certain non-radicals that either are oxidizing agents or are easily converted into radicals or both (Biswas 2016). Recent studies have demonstrated that reducing the production of ROS *via* eucalyptol or ROS scavengers significantly alleviated pain and inflammation in a mouse with gout, demonstrating the key role of ROS in mediating gout pain and inflammation (Yin et al., 2020a; Yin et al., 2020b). Besides, it has been shown that arachidonic acid can promote the production of ROS (Shin and Kim 2009). Several components have been shown to play antioxidative roles by reducing the levels of ROS , such as astilbin (peak 103) (Wang et al., 2018) and engeletin (peak 111) (Zhao et al., 2020). Therefore, TDFC may partially exert its anti-gout effect by decreasing ROS formation.

Nitric oxide (NO), a small gas molecule, is synthesized by three isoforms of NO synthase (NOS): endothelial NOS, neuronal NOS, and inducible NOS (iNOS) (Michel and Feron 1997). Among them, the overexpression of iNOS is linked to a variety of pathological conditions, including gouty arthritis (Chen et al., 2004), and inhibition of iNOS attenuates MSU-induced inflammation in mice (Ju et al., 2011). In addition, an earlier study has shown that, in pathological conditions, arachidonic acid can stimulate the expression of NO (Mariotto et al., 2007). Several components have been shown to effectively inhibit NOS, such as magnoflorine (peak 30) (Wang et al., 2019), menisperine (peak 42) (Zhao et al., 2020), cyasterone (peak 94) (Cao et al., 2017), astilbin (peak 103) (Zhao et al., 2020), engeletin (peak 111) (Ko et al., 2004), and apigenin (peak 130) (Ko et al., 2004). Therefore, TFDC may play an anti-gout role by inhibiting NOS expression.

In summary, by acting on multiple target genes/proteins, or genes/proteins associated with multiple pathways, the multiple constituents contained in TFDC exert their therapeutic effects on gout. However, there are some unavoidable limitations to this study. Firstly, in some situations, it is the metabolites rather than the parent compounds that possess therapeutic effects. Secondly, the effects and targets of chemicals are likely tissue- and dose-specific, which may affect the accuracy of our predicted results. Thirdly, it is indeed difficult to confirm the predicted targets with inhibitory or activating effects *in vivo*. Fourthly, the constituents in TFDC were treated equally, without considering their contents. Finally, and importantly, the theoretical predictions may be influenced by potential biases in the highly studied biological processes (Yu et al., 2019).

### 3.4 Real-Time PCR Assay

**FIGURE 8:**
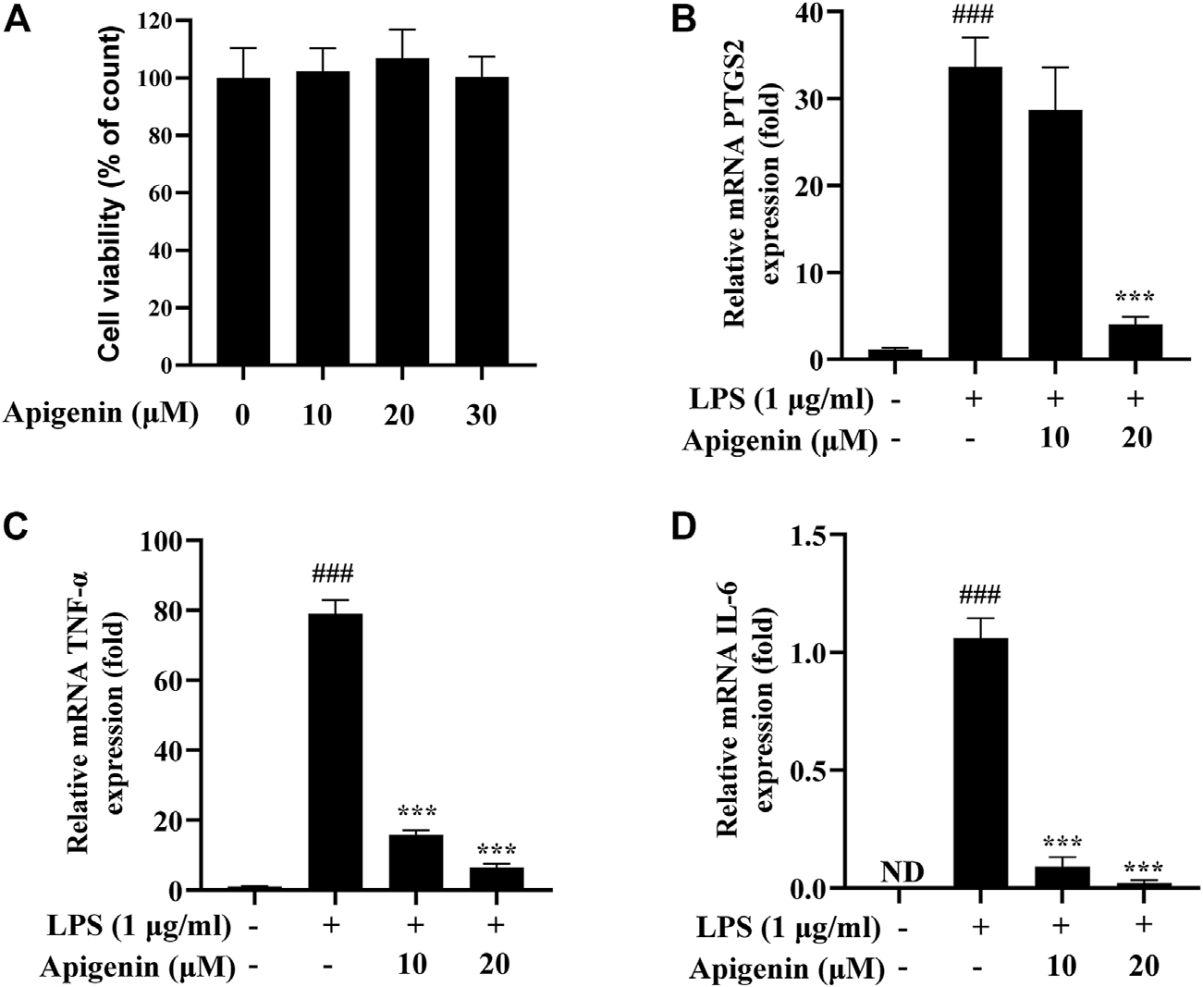
Tohoku Hospital Pediatrics-1 (THP-1) macrophages were treated with apigenin and lipopolysaccharide (LPS; 1 μg/ml) and subsequently incubated for 18 h. The effects of apigenin on the mRNA expressions of prostaglandin G/H synthase 2 (PTGS2), tumor necrosis factor alpha (TNF-α), and interleukin 6 (IL-6) are shown. **(A)** Cell viability as determined by the CCK-8 assay. **(B–D)** The mRNA expressions of PTGS2 **(B)**, TNF-α **(C)**, and IL-6 **(D)** were detected by real-time PCR. Data are the mean ± SD from three separate experiments. *ND*, not detected. ^###^ *p* < 0.001 *versus* control; ***p < 0.001 *versus* treatment with LPS alone.

Real-time PCR experiment was used to verify the effects of the predicted active components on the predicted targets. To select suitable doses and evaluate the effect of apigenin on the LPS-induced THP-1 cell model, the cytotoxicity of apigenin was examined. As shown in Figure 8A, the concentration of apigenin from 10 to 30 μM showed no effect on cell growth. Hence, apigenin doses of 10 μM (low dose) and 20 μM (high dose) were chosen as the doses for subsequent experiments. The real-time PCR experiment results, presented in Figures 8B–D, showed that the mRNA expression levels of PTGS2, TNF-α, and IL-6 in the LPS group were significantly increased compared with those in the control group. Meanwhile, the mRNA productions of PTGS2, TNF-α, and IL-6 were remarkably reduced after treatment with apigenin. These results preliminarily indicate that apigenin could modulate the pathway of arachidonic acid.

## 4 Conclusion

Compared with conventional screening of absorbed compounds in partial site, we developed a novel strategy for stepwise screening from *in vitro*, *in situ*, to *in vivo* by integrating UPLC-MS and IPVS to characterize the multi-site absorption process of TCM. Using this strategy, firstly, we described the chemical profile of TFDC rapidly and systematically by identifying and characterizing 141 components. Then, 65 absorbed prototype compounds were rapidly identified by combining the use of the IPVS and IG methods. Next, a target network pharmacology analysis was performed based on the identified absorbed components, and the results indicated that the arachidonic acid metabolism pathway was highly enriched and that eight key targets were found, suggesting that the mechanism of action of TFDC in treating gout may be mainly *via* regulating the arachidonic acid metabolism pathway. Moreover, the real-time PCR experiment showed that apigenin can suppress the mRNA expressions of PTGS2, TNF-α, and IL-6 in LPS-induced THP-1 cells. Collectively, the results demonstrated that this novel strategy may be a powerful tool to rapidly screen potential active components from TCM and clarify the mechanism of action of the active compounds in the treatment of diseases to support assessments of potential clinical application.

## Data Availability

The original contributions presented in the study are included in the article/Supplementary Material. Further inquiries can be directed to the corresponding authors.
