# Supplementary material for: Integrated Strategy From In Vitro, In Situ, In Vivo to In Silico for Predicting Active Constituents and Exploring Molecular Mechanisms of Tongfengding Capsule for Treating Gout by Inhibiting Inflammatory Responses
Source: Front Pharmacol. 2021 Nov 29;12:759157. doi: 10.3389/fphar.2021.759157 (PMC8666879; doi:10.3389/fphar.2021.759157)
Supplement: Supplementary file 1 [file DataSheet3.docx]

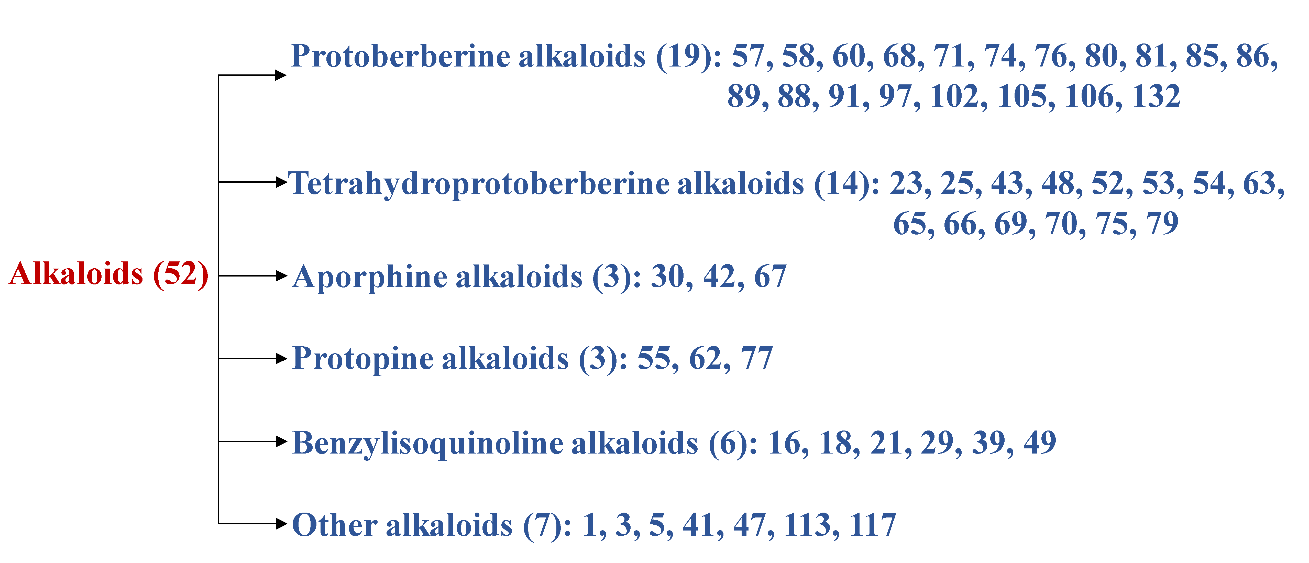


**Supplementary Figure 1.** Alkaloids identified in TFDC by UHPLC-Q Exactive-Orbitrap HRMS.

**Supplementary Figure 2.** MS^2^ spectra and proposed fragmentations for berberine (88) from TFDC.

**Supplementary Figure 3.** MS^2^ spectra and proposed fragmentations for tetrahydropalmatine (66) from TFDC.

**Supplementary Figure 4.** MS^2^ spectra and proposed fragmentations for magnoflorine (30) from TFDC.

**Supplementary Figure 5.** MS^2^ spectra and proposed fragmentations for protopine (55) from TFDC.

**Supplementary Figure 6.** MS^2^ spectra and proposed fragmentations for magnocurarine (16) from TFDC

**Supplementary Figure 7.** MS^2^ spectra and proposed fragmentations for astilbin (103) from TFDC.


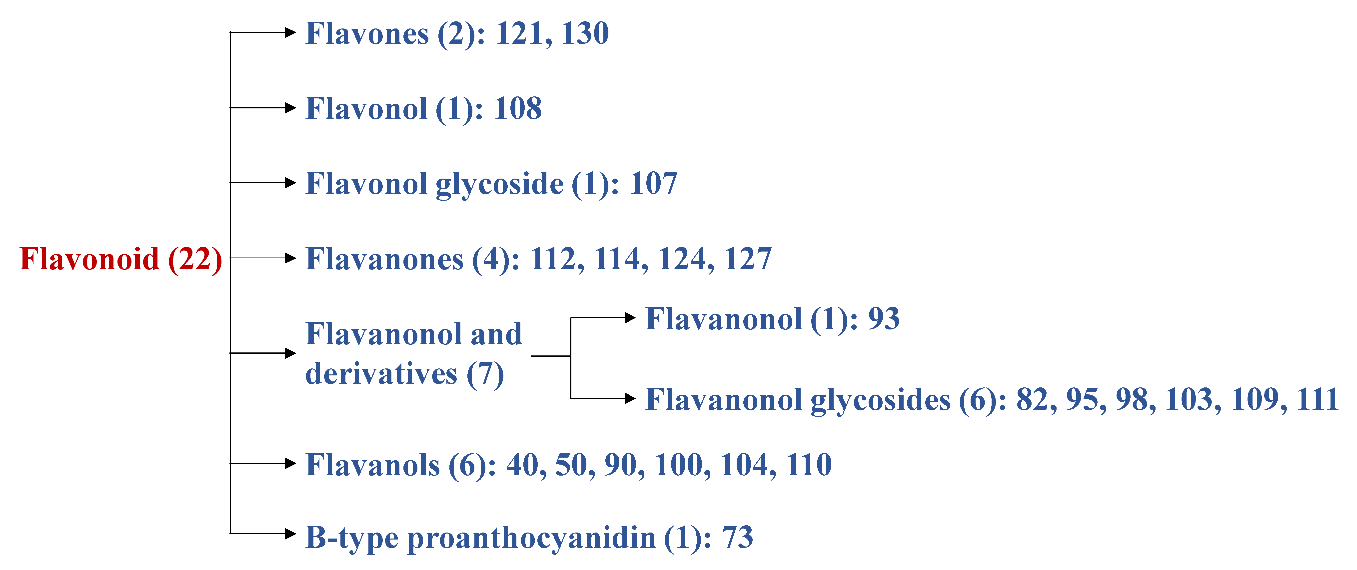


**Supplementary Figure 8.** Flavonoids identified in TFDC by UHPLC-Q Exactive-Orbitrap HRMS.


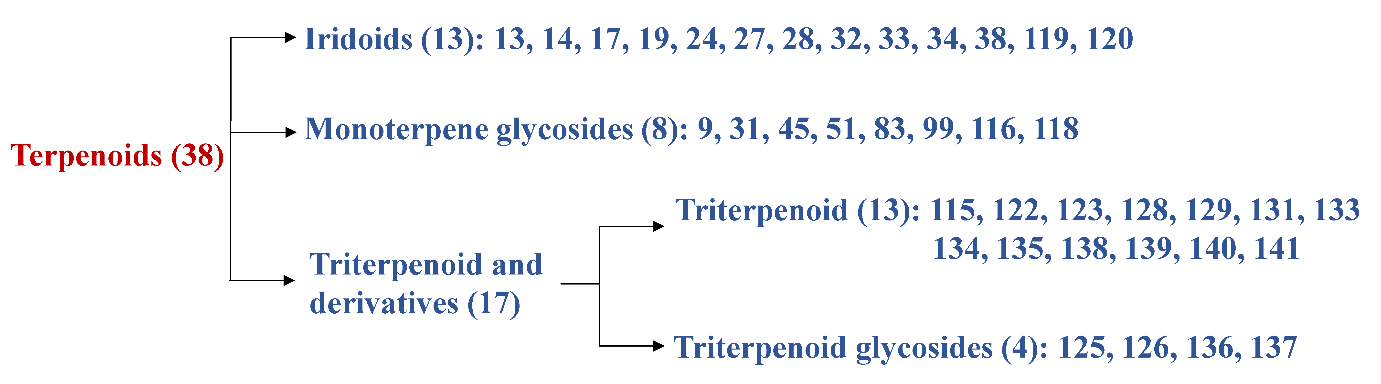


**Supplementary Figure 9.** Terpenoids identified in TFDC by UHPLC-Q Exactive-Orbitrap HRMS.

**Supplementary Figure 10.** MS^2^ spectra and proposed fragmentations for loganic acid (19) from TFDC.

**Supplementary Figure 11.** MS^2^ spectra and proposed fragmentations for oxypaeoniflorin (31) from TFDC.

**Supplementary Figure 12.** MS^2^ spectra and proposed fragmentations for 16-oxoalisol A (122) from TFDC.


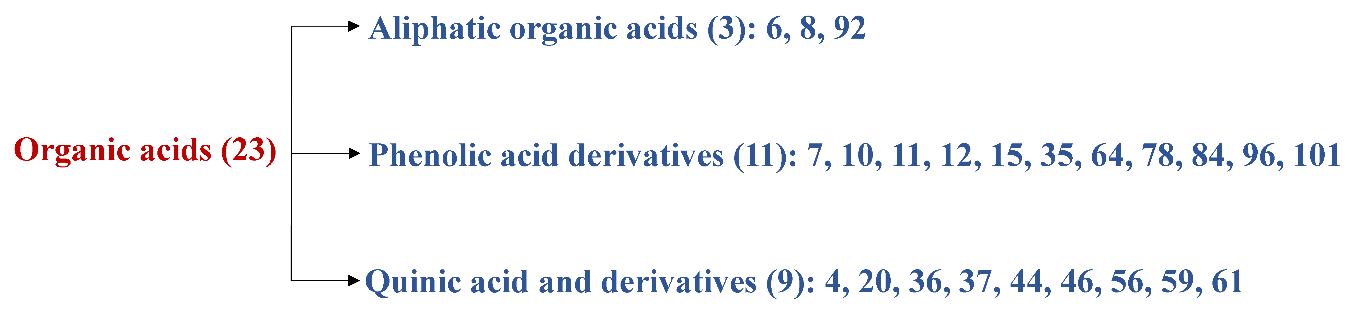


**Supplementary Figure 13.** Organic acids identified in TFDC by UHPLC-Q Exactive-Orbitrap HRMS.

**Supplementary Figure 14.** MS^2^ spectra and proposed fragmentations for 1'-O-galloylsucrose (7) from TFDC.

**Supplementary Figure 15.** MS^2^ spectra and proposed fragmentations for cryptochlorogenic acid (37) from TFDC.
