# Supplementary material for: Integrated Strategy From In Vitro, In Situ, In Vivo to In Silico for Predicting Active Constituents and Exploring Molecular Mechanisms of Tongfengding Capsule for Treating Gout by Inhibiting Inflammatory Responses
Source: Front Pharmacol. 2021 Nov 29;12:759157. doi: 10.3389/fphar.2021.759157 (PMC8666879; doi:10.3389/fphar.2021.759157)
Supplement: Supplementary file 2 [file DataSheet1.docx]

**Supplementary Table 1.** Identification of Compounds in TFDC by UHPLC-Q Exactive-Orbitrap HRMS in positive ion mode.

| peak no. | *t*_R_  (min) | molecular  formula | measured  mass  [M+H]^+^ | error  (ppm) | MS/MS fragments (*m/z*) | identification  compound | source |
| --- | --- | --- | --- | --- | --- | --- | --- |
| 1* | 0.73 | C_5_H_5_N_5_ | 136.0617 | 0.058 | 119.0354[M+H-NH_3_]^+^, 94.0404[M+H-NCHNH]^+^ | adenine | PS |
| 3* | 0.83 | C_10_H_13_N_5_O_4_ | 268.1032 | -0.669 | 136.0618[M+H-C_5_H_8_O_4_]^+^ | adenosine | PS |
| 5 | 0.86 | C_6_H_5_NO_2_ | 124.0395 | -0.313 | 80.0500[M+H-CO_2_]^+^ | nicotinic acid | PS |
| 16 | 3.48 | C_19_H_24_NO_3_^+^ | 314.1748[M]^+^ | -0.955 | 298.1064[M-CH_4_]^+^, 269.1172[M-(CH_3_)_2_NH]^+^, 237.0900[M-(CH_3_)_2_NH-CH_3_OH]^+^, 107.0494[M-C_12_H_16_NO_2_]^+^ | magnocurarine | PC |
| 18 | 3.77 | C_19_H_24_NO_3_^+^ | 314.1748[M]^+^ | -0.955 | 298.1064[M-CH_4_]^+^, 269.1172[M-(CH_3_)_2_NH]^+^, 237.0900[M-(CH_3_)_2_NH-CH_3_OH]^+^, 107.0494[M-C_12_H_16_NO_2_]^+^ | lotusine | PC |
| 21 | 3.99 | C_18_H_20_NO_3_^+^ | 298.1436[M]^+^ | -0.738 | 283.1198[M-CH_3_]^+^, 255.1238[M-CH_3_-CO]^+^ | A_1_ | PC |
| 23 | 4.29 | C_20_H_22_NO_5_^+^ | 356.1487[M]^+^ | -1.430 | 206.0810[M-C_9_H_10_O_2_] ^+^, 191.0575[M-C_9_H_10_O_2_-CH_3_]^+^ | derivative of phellodendrine | PC |
| 25* | 4.42 | C_20_H_24_NO_4_^+^ | 342.1696[M]^+^ | -1.241 | 192.1017[M-C_9_H_10_O_2_]^+^, 177.0783[M-C_9_H_10_O_2_-CH_3_]^+^ | phellodendrine | PC |
| 28 | 4.58 | C_22_H_30_O_14_ | 541.1528  [M+Na]^+^ | -0.012 | 365.1051[M+Na-C_10_H_8_O_3_]^+^, 347.0944[M+Na-C_10_H_8_O_3_-H_2_O]^+^ | 6'-O-*β*-D-glucosylgentiopicroside | GM |
| 29 | 4.84 | C_23_H_29_NO_8_ | 448.1963 | -0.744 | 286.1433[M+H-Glc]^+^, 255.1013[M+H-Glc-CH_3_NH_2_]^+^, 107.0794[C_7_H_7_O]^+^ | N-methylhigenamine 7-  glucopyranoside | PC |
| 30* | 4.89 | C_20_H_24_NO_4_^+^ | 342.1696 | -1.154 | 297.1117[M-(CH_3_)_2_NH]^+^, 282.0883[M-(CH_3_)_2_NH-CH_3_]^+^, 265.0856[M-(CH_3_)_2_NH-CH_3_OH]^+^, 237.0907[M-(CH_3_)_2_NH-CH_3_OH-CO]^+^ | magnoflorine | PC |
| 33 | 5.19 | C_22_H_30_O_14_ | 541.1523  [M+Na]^+^ | -0.918 | 365.1051[M+Na-C_10_H_8_O_3_]^+^, 347.0944[M+Na-C_10_H_8_O_3_-H_2_O]^+^ | 4'-O-*β*-D-glucosylgentiopicroside | GM |
| 38 | 5.49 | C_22_H_30_O_14_ | 541.1526  [M+Na]^+^ | -0.234 | 365.1051[M+Na-C_10_H_8_O_3_]^+^, 347.0944[M+Na-C_10_H_8_O_3_-H_2_O]^+^ | olivieroside C | GM |
| 39 | 5.52 | C_19_H_24_NO_3_^+^ | 314.1748[M]^+^ | -0.955 | 298.1064[M-CH_4_]^+^, 269.1172[M-(CH_3_)_2_NH]^+^, 237.0900[M-(CH_3_)_2_NH-CH_3_OH]^+^, 107.0494[M-C_12_H_16_NO_2_]^+^ | oblongine | PC |
| 41 | 5.57 | C_11_H_19_N_3_O_2_ | 226.1549 | -0.634 | 208.1443[M+H-H_2_O]^+^, 84.0561[M+H-C_8_H_14_O_2_]^+^ | plantagoguanidinic acid | PS |
| 42 | 5.70 | C_21_H_26_NO_4_^+^ | 356.1852[M]^+^ | -1.277 | 311.1270[M-(CH_3_)_2_NH]^+^, 296.1035[M-(CH_3_)_2_NH-CH_3_]^+^  279.1010[M-(CH_3_)_2_NH-CH_3_OH]^+^, 264.0777[M-(CH_3_)_2_NH-CH_3_-CH_3_OH]^+^ | menisperine | PC |
| 43 | 5.80 | C_19_H_21_NO_4_ | 328.1544 | 0.169 | 313.1308[M+H-CH_3_]^+^, 178.0858[M+H-C_9_H_10_O_2_]^+^,  163.0623[M+H-C_9_H_10_O_2_-CH_3_]^+^, 151.0749[M+H-C_10_H_11_NO_2_]^+^ | scoulerine | CR |
| 47 | 6.01 | C_17_H_21_NO_4_ | 304.1543 | -0.114 | 286.1426[M+H-H_2_O]^+^, 271.1191[M+H-H_2_O-CH_3_]^+^,  232.0960[M+H-H_2_O-C_4_H_6_]^+^, 202.0857[M+H-C_4_H_8_O-CH_2_O]^+^ | dasycarpamin | PC |
| 48 | 6.03 | C_20_H_24_NO_4_^+^ | 342.1695[M]^+^ | -1.329 | 192.1017[M-C_9_H_10_O_2_]^+^, 177.0783[M-C_9_H_10_O_2_-CH_3_]^+^ | phellodendrine isomer | PC |
| 49 | 6.19 | C_20_H_26_NO_4_^+^ | 344.1851[M]^+^ | -0.159 | 299.1266[M-(CH_3_)_2_NH]^+^, 206.1171[M-C_8_H_10_O_2_]^+^,  175.0750[M-C_9_H_15_NO_2_]^+^, 137.0594[M-C_12_H_17_NO_2_]^+^ | tembetarine | PC |
| 52 | 6.63 | C_21_H_25_NO_4_ | 356.1851 | -1.445 | 340.1539[M+H-CH_4_]^+^, 192.1018[M+H-C_9_H_10_O_2_]^+^,  177.0784[M+H-C_9_H_10_O_2_-CH_3_]^+^, 165.0909[M+H-C_11_H_13_NO_2_]^+^ | yuanhunine or its isomer | CR |
| 53 | 6.67 | C_20_H_24_NO_4_^+^ | 342.1698[M]^+^ | -0.452 | 326.1380[M-CH_4_]^+^, 192.1018[M-C_9_H_11_O_2_] ^+^, 178.0862[M-C_10_H_13_O_2_]^+^, 163.0627[M-C_10_H_13_O_2_-CH_3_] ^+^, 151.0755[M-C_11_H_14_NO_2_-CH_3_]^+^ | tetrahydrocolumbamine | CR |
| 54 | 6.81 | C_21_H_25_NO_4_ | 356.1851 | -1.445 | 340.1531[M+H-CH_4_]^+^, 192.1018[M+H-C_9_H_10_O_2_]^+^, 177.0784[M+H-C_9_H_10_O_2_-CH_3_]^+^, 165.0909[M+H-C_11_H_13_NO_2_]^+^ | yuanhunine or its isomer | CR, PC |
| 55* | 7.05 | C_20_H_19_NO_5_ | 354.1331 | -1.353 | 336.1231[M+H-H_2_O]^+^, 206.0812[M+H-C_9_H_8_O_2_]^+^，188.0705[M+H-C_9_H_8_O_2_-H_2_O]^+^,189.0783[M+H-C_9_H_8_O_2_-OH]^+^,  165.0545[M+H-C_11_H_11_NO_2_]^+^, 149.0597[M+H-C_11_H_11_NO_3_]^+^ | protopine | CR |
| 57 | 7.15 | C_20_H_18_NO_5_^+^ | 352.1173[M]^+^ | -1.900 | 336.0863[M-CH_4_]^+^, 337.0928[M-CH_3_]^+^, 322.0706[M-CH_3_-CH_3_]^+^, 308.0913[M-CH_4_-CO]^+^, 294.0755[M-CH_3_-CH_3_-CO]^+^ | berberastine | PC |
| 58 | 7.34 | C_19_H_18_NO_4_^+^ | 324.1229[M]^+^ | -0.446 | 308.0913[M-CH_4_]^+^, 294.0758[M-CH_3_-CH_3_]^+^, 280.0965[M-CH_4_-CO]^+^, 266.0809[M-CH_3_-CH_3_-CO]^+^ | demethyleneberberine | PC |
| 60 | 7.58 | C_19_H_18_NO_4_^+^ | 324.1231[M]^+^ | 0.233 | 309.0990[M-CH_3_]^+^, 294.0756[M-CH_3_-CH_3_]^+^, 280.0966[M-CH_4_-CO]^+^, 266.0807[M-CH_3_-CH_3_-CO]^+^ | demethyleneberberine isomer | PC |
| 62 | 7.71 | C_21_H_23_NO_5_ | 370.1643 | -1.673 | 352.1537[M+H-H_2_O]^+^, 336.1223[M+H-H_2_O-CH_4_]^+^, 206.0807[M+H-C_10_H_12_O_2_]^+^, 188.0701[M+H-C_10_H_12_O_2_-H_2_O]^+^, 149.0596[M+H-C_12_H_15_NO_3_]^+^ | allocryptopine | CR |
| 63 | 7.75 | C_21_H_25_NO_4_^+^ | 356.1855 | -0.491 | 340.1537[M+H-CH_4_]^+^, 178.0862[M+H-C_11_H_14_O_2_]^+^, 163.0629[M+H-C_11_H_14_O_2_-CH_3_]^+^, 151.0754[M+H-C_12_H_15_NO_2_]^+^ | isocorybulbine | CR |
| 65 | 7.90 | C_22_H_28_NO_4_^+^ | 370.2008[M]^+^ | -1.283 | 354.1692[M-CH_4_]^+^, 206.1174[M-C_10_H_12_O_2_]^+^, 190.0861[M-C_10_H_12_O_2_-CH_4_]^+^, 165.0910[M-C_12_H_15_NO_2_]^+^ | N-methyltetrahydropalmatine | PC, CR |
| 66* | 7.98 | C_21_H_25_NO_4_ | 356.1849 | -2.035 | 340.1540[M+H-CH_4_]^+^, 192.1017[M+H-C_10_H_12_O_2_]^+^, 177.0782[M+H-C_10_H_12_O_2_-CH_3_]^+^, 165.0909[M+H-C_11_H_13_NO_2_]^+^, 150.0674[M+H-C_11_H_13_NO_2_-CH_3_]^+^ | tetrahydropalmatine | CR |
| 67 | 8.07 | C_21_H_25_NO_4_ | 356.1854 | -0.771 | 325.1431[M+H-CH_3_NH]^+^, 310.1195[M+H-CH_3_NH-CH_3_]^+^, 294.1245[M+H-CH_3_NH-  OCH_3_]^+^, 279.1012[M+H-CH_3_NH-OCH_3_-CH_3_]^+^, 251.1063[M-CH_3_NH-OCH_3_-CH_3_-CO]^+^ | glaucine | CR |
| 68* | 8.16 | C_19_H_14_NO_4_^+^ | 320.0917[M]^+^ | 0.017 | 292.0964[M-CO]^+^, 277.0732[M-CO-CH_3_]^+^, 262.0860[M-2CO-2H]^+^, 234.0911[M-3CO-2H]^+^ | coptisine | CR |
| 69 | 8.16 | C_19_H_17_NO_4_ | 324.1227 | -1.186 | 176.0705[M+H-C_9_H_8_O_2_]^+^, 149.0597[M+H-C_10_H_9_NO_2_]^+^ | tetrahydrocoptisine | CR |
| 70 | 8.34 | C_21_H_24_NO_4_^+^ | 354.1700[M]^+^ | 0.071 | 338.1381[M-CH_4_]^+^, 190.0861[M-C_10_H_12_O_2_]^+^, 165.0909[M-C_11_H_11_NO_2_]^+^ | N-methylcanadine | CR |
| 71* | 8.37 | C_20_H_20_NO_4_^+^ | 338.1384[M]^+^ | -0.753 | 323.1143[M-CH_3_]^+^, 322.1070[M-CH_4_]^+^, 308.0906[M-2CH_3_]^+^, 294.1116[M-CH_4_-CO]^+^, 280.0957[M-2CH_3_-CO]^+^ | columbamine | PC, CR |
| 74* | 8.59 | C_20_H_20_NO_4_^+^ | 338.1384[M]^+^ | -0.930 | 323.1143[M-CH_3_]^+^, 322.1070[M-CH_4_]^+^, 308.0906[M-2CH_3_]^+^, 294.1116[M-CH_4_-CO]^+^, 280.0957[M-2CH_3_-CO]^+^ | jatrorrhizine | PC, CR |
| 75* | 8.67 | C_20_H_21_NO_4_ | 340.1539 | -1.190 | 324.1223[M+H-CH_4_]^+^, 176.0704[M+H-C_10_H_12_O_2_]^+^, 165.0907[M+H-C_11_H_9_NO_2_]^+^ | tetrahydroberberine | CR |
| 76 | 8.70 | C_19_H_15_NO_4_ | 322.1072 | -0.635 | 307.0834[M+H-CH_3_]^+^, 279.0887[M+H-CH_3_-CO]^+^ | thalifendine | PC |
| 77 | 8.70 | C_21_H_23_NO_5_ | 370.1646 | -0.701 | 352.1527[M+H-H_2_O]^+^, 190.0857[M+H-C_10_H_12_O_3_]^+^, 149.0594[M+H-C_12_H_15_NO_3_]^+^ | cryptopine | CR |
| 79* | 8.97 | C_22_H_27_NO_4_ | 370.2008 | -1.202 | 354.1699[M+H-CH_4_]^+^, 192.1017[M+H-C_11_H_14_O_2_]^+^, 179.1065[M+H-C_11_H_12_NO_2_]^+^, 165.0909[M+H-C_11_H_12_NO_2_-CH_2_]^+^ | corydaline | CR |
| 80 | 9.09 | C_20_H_16_NO_4_^+^ | 334.1069[M]^+^ | -1.450 | 319.0842[M-CH_3_]^+^, 306.1118[M-CO]^+^, 291.0881[M-CH_3_-CO]^+^, 276.1014[M-2CO-2H]^+^ | corysamine | CR |
| 81 | 9.12 | C_21_H_22_NO_4_^+^ | 352.1541[M]^+^ | -0.723 | 336.1226[M-CH_4_]^+^, 322.1065[M-2CH_3_]^+^, 308.1277[M-CH_4_-CO]^+^, 294.1112[M-2CH_3_-CO]^+^ | dehydrocorybulbine | PC, CR |
| 85 | 9.22 | C_19_H_15_NO_4_ | 322.1074 | -0.076 | 307.0834[M+H-CH_3_]^+^, 292.0604[M+H-CH_3_-CH_3_]^+^, 279.0884[M+H-CH_3_-CO]^+^, 264.0658[M+H-CH_3_-CH_3_-CO]^+^, 251.0937[M+H-CH_3_-CO-CO]^+^ | berberrubine | PC |
| 86 | 9.28 | C_21_H_22_NO_4_^+^ | 352.1538[M]^+^ | -1.490 | 337.1300[M-CH_3_]^+^, 322.1060[M-2CH_3_]^+^, 308.1273[M-CH_4_-CO]^+^, 293.1042[M-CH_4_-CO-CH_3_]^+^ | 13-methyldehydrocorydalmine | CR |
| 88* | 9.53 | C_20_H_18_NO_4_^+^ | 336.1226[M]^+^ | -1.412 | 320.0914[M-CH_4_]^+^, 306.0757[M-2CH_3_]^+^, 292.0963[M-CH_4_-CO]^+^, 278.0808[M-2CH_3_-CO]^+^ | berberine | PC, CR |
| 89* | 9.53 | C_21_H_22_NO_4_^+^ | 352.1537[M]^+^ | -1.831 | 336.1226[M-CH_4_]^+^, 322.1065[M-2CH_3_]^+^, 308.1275[M-CH_4_-CO]^+^, 294.1118[M-2CH_3_-CO]^+^ | palmatine | PC, CR |
| 91 | 9.80 | C_21_H_22_NO_4_^+^ | 352.1541[M]^+^ | -0.723 | 337.1299[M-CH_3_]^+^, 322.1060[M-2CH_3_]^+^, 308.1270[M-CH_4_-CO]^+^, 293.1037[M-CH_4_-CO-CH_3_]^+^ | 13-methylpalmatrubine | CR |
| 97* | 10.23 | C_22_H_24_NO_4_^+^ | 366.1691[M]^+^ | -2.498 | 350.1384[M-CH_4_]^+^, 336.1222[M-2CH_3_]^+^, 322.1433[M-CH_4_-CO]^+^,  308.1276[M-2CH_3_-CO]^+^ | dehydrocorydaline | CR |
| 102 | 10.63 | C_22_H_24_NO_4_^+^ | 366.1696[M]^+^ | -1.078 | 351.1457[M-CH_3_]^+^, 336.1226[M-2CH_3_]^+^, 321.0990[M-CH_3_-CO-2H]^+^, 308.1258[M-2CH_3_-CO]^+^ | dehydrocorydaline isomer | CR |
| 105 | 11.52 | C_29_H_28_NO_6_^+^ | 486.1911[M]^+^ | -0.091 | 336.1225[M-C_9_H_10_O_2_]^+^, 320.0914[M-C_9_H_10_O_2_-CH_4_]^+^, 306.0757[M-C_9_H_10_O_2_-2CH_3_]^+^,  292.0963[M-C_9_H_10_O_2_-CH_4_-CO]^+^, 278.0807[M-C_9_H_10_O_2_-2CH_3_-CO]^+^ | derivative of berberine | PC |
| 106 | 11.64 | C_29_H_28_NO_6_^+^ | 486.1911[M]^+^ | -0.029 | 336.1227[M-C_9_H_10_O_2_]^+^, 320.0914[M-C_9_H_10_O_2_-CH_4_]^+^, 306.0757[M-C_9_H_10_O_2_-2CH_3_]^+^,  292.0964[M-C_9_H_10_O_2_-CH_4_-CO]^+^, 278.0811[M-C_9_H_10_O_2_-2CH_3_-CO]^+^ | derivative of berberine | PC |
| 108* | 11.70 | C_15_H_10_O_7_ | 303.0499 | 0.003 | 257.0442[M+H-CH_2_O_2_]^+^, 229.0494[M+H-C_2_H_2_O_3_]^+^, 153.0182[M+H-C_8_H_6_O_3_]^+^, 137.0234[M+H-C_8_H_6_O_4_]^+^ | quercetin | SG |
| 113 | 13.85 | C_14_H_13_NO_4_ | 260.0918 | 0.367 | 245.0682[M+H-CH_3_]^+^, 227.0576[M+H-CH_3_-H_2_O]^+^, 216.0656[M+H-C_2_H_4_O]^+^,  199.0627227.0576[M+H-CH_3_-H_2_O-CO]^+^, 184.0392[M+H-CH_3_-H_2_O-CO-CH_3_]^+^ | skimmianine | PC |
| 117 | 14.54 | C_13_H_11_NO_3_ | 230.0811 | 0.349 | 215.0570[M+H-CH_3_]^+^, 200.0336[M+H-2CH_3_]^+^, 186.0544[M+H-C_2_H_4_O]^+^, 172.0389[M+H-2CH_3_-CO]^+^ | γ-fagarine | PC |
| 122 | 15.79 | C_30_H_48_O_6_ | 505.3524 | 0.048 | 487.3419[M+H-H_2_O]^+^, 469.3312[M+H-2H_2_O]^+^, 451.3202[M+H-3H_2_O]^+^, 415.2842[M+H-C_4_H_10_O_2_]^+^, 397.2735[M+H-C_4_H_10_O_2_-H_2_O]^+^,  353.2471[M+H-C_4_H_10_O_2_-H_2_O-C_2_H_4_O]^+^ | 16-oxoalisol A | AR |
| 128* | 16.35 | C_26_H_30_O_8_ | 471.2013 | -2.365 | 425.1946[M+H-CH_2_O_2_]^+^, 367.1888[M+H-C_3_H_4_O_5_]^+^, 213.0904[M+H-C_12_H_18_O_6_]^+^, 161.0592[C_10_H_9_O_2_]^+^, 133.0643[C_9_H_9_O]^+^ | limonin | PC |
| 129 | 16.59 | C_26_H_32_O_8_ | 473.2161 | -1.911 | 455.2052[M+H-H_2_O]^+^, 427.2105[M+H-H_2_O-CO]^+^, 369.2053[M+H-C_3_H_4_O_4_]^+^, 161.0593[C_10_H_9_O_2_]^+^ | obacunoic acid | PC |
| 132 | 16.93 | C_20_H_18_NO_5_ | 352.1176 | -1.134 | 337.0940[M-CH_3_]^+^, 322.0706[M-2CH_3_]^+^, 308.0913[M-C_2_H_4_O]^+^,  294.0757[M-2CH_3_-CO]^+^ | derivative of jatrorrhizine | PC |
| 133 | 16.96 | C_30_H_46_O_5_ | 487.3416 | -0.515 | 469.3310[M+H-H_2_O]^+^, 451.3204[M+H-2H_2_O]^+^, 397.2733[M+H-C_4_H_10_O_2_]^+^, 353.2472[M+H-C_4_H_10_O_2_-C_2_H_4_O]^+^ | 11-anhydro-16-oxoalisol A | AR |
| 134 | 17.01 | C_30_H_48_O_5_ | 489.3574 | -0.125 | 471.3469[M+H-H_2_O]^+^, 453.3360[M+H-2H_2_O]^+^, 399.2894[M+H-C_4_H_10_O_2_]^+^ , 355.2626[M+H-C_4_H_10_O_2_-C_2_H_4_O]^+^ | 11-Deoxy-16-oxoalisol A | AR |
| 135 | 17.20 | C_26_H_30_O_7_ | 455.2062 | -0.549 | 437.1946[M+H-H_2_O]^+^, 409.2002[M+H-CH_2_O_2_]^+^, 161.0592[C_9_H_10_O_2_]^+^, 105.0700[C_8_H_9_]^+^ | obacunone | PC |
| 138 | 17.51 | C_32_H_48_O_6_ | 529.3524 | 0.103 | 511.3424[M+H-H_2_O]^+^, 469.3315[M+H-C_2_H_4_O]^+^,  451.3205[M+H-C_2_H_4_O-H_2_O]^+^, 415.2841[M+H-C_2_H_4_O-C_4_H_6_]^+^,  353.2473[M+H-C_2_H_4_O-C_4_H_6_-H_2_O-C_2_H_4_O]^+^ | alisol C 23-acetate or its isomer | AR |
| 139 | 17.52 | C_30_H_46_O_4_ | 471.3465 | -0.820 | 453.3400[M+H-H_2_O]^+^, 381.2780[M+H-C_4_H_10_O_2_]^+^, 339.2761[M+H-C_4_H_10_O_2_-C_2_H_4_O]^+^ | 24-deacetyl-alisolO/  16,23-oxido-alisol B | AR |
| 140 | 18.21 | C_32_H_50_O_5_ | 515.3732 | -0.814 | 497.3636[M+H-H_2_O]^+^, 437.3418[M+H-C_2_H_6_O_3_]^+^, 419.3298[M+H-C_2_H_6_O_3_-H_2_O]^+^,  383.2943[M+H-H_2_O-C_6_H_10_O_2_]^+^, 365.2837[M+H-H_2_O-C_6_H_10_O_2_]^+^,  339.2679[M+H-H_2_O-C_8_H_14_O_3_]^+^ | 25-dehydroxy alisol A 24-acetate | AR |
| 141 | 19.13 | C_32_H_50_O_5_ | 515.3730 | -1.163 | 455.3489[M+H-C_2_H_4_O_2_]^+^, 437.3424[M+H-C_2_H_6_O_3_]^+^, 419.3315[M+H-C_2_H_6_O_3_-H_2_O]^+^, 383.2914[M+H-H_2_O-C_6_H_10_O_2_]^+^, 365.2837[M+H-H_2_O-C_6_H_10_O_2_]^+^,  339.2671[M+H-H_2_O-C_8_H_14_O_3_]^+^ | alisol B 23-acetate | AR |

Note: *t*_R_: retention time; *: compound identified by compared with a reference standard; Glc: *β*-D-glucose; GM: *Gentianae Macrophyllae* Radix; PC: *Phellodendri Chinensis* Cortex;

CR: *Corydalis* Rhizoma; PR: *Paeoniae* Radix Rubra; CTR: *Cyathulae* Radix; AR: *Alismatis* Rhizoma; PS: *Plantaginis* Semen; SG: *Smilacis Glabrae* Rhizoma.

A_1_: 3,4-Dihydro-1-[(4-hydroxyphenyl)methyl]-7-methoxy-2-methyl-6-isoquinolinol.

**Supplementary Table 2.** Identification of Compounds in TFDC by UHPLC-Q Exactive-Orbitrap HRMS in negative ion mode.

| peak no. | *t*_R_  (min) | molecular  formula | measured  mass  [M-H]^-^ | error  (ppm) | MS/MS fragments (*m/z*) | identification  compound | source |
| --- | --- | --- | --- | --- | --- | --- | --- |
| 2 | 0.78 | C_12_H_22_O_11_ | 341.1082 | -0.399 | 179.0552[M-H-C_6_H_10_O_5_]^-^,161.0444[M-H-C_6_H_10_O_5_-H_2_O]^-^,  119.0338[M-H-C_6_H_10_O_5_-CH_2_OH-CHO]^-^, 89.0231[M-H-C_6_H_10_O_5_-O-CH_2_OH-CHOH]^-^ | sucrose | all |
| 4 | 0.84 | C_7_H_12_O_6_ | 191.0551 | -1.324 | 173.0450[M-H-H_2_O]^-^ | quinic acid | PC |
| 6 | 0.90 | C_4_H_6_O_5_ | 133.0131 | -4.647 | 115.0024 [M-H-H_2_O]^-^, 89.0231[M-H-CO_2_]^-^, 71.0125[M-H-CO_2_-H_2_O]^-^ | malic acid | all |
| 7 | 0.92 | C_19_H_26_O_15_ | 493.1197 | 1.812 | 331.0665[M-H-Glc]^-^, 313.0565[M-H-Glc-H_2_O]^-^, 169.0133[M-H-C_12_H_20_O_10_]^-^,  125.0232[M-H-C_12_H_20_O_10_-CO_2_]^-^ | 1'-O-galloylsucrose | PR |
| 8 | 1.09 | C_6_H_8_O_7_ | 191.0188 | -2.238 | 173.0084[M-H-H_2_O]^-^, 129.0182[M-H-H_2_O-CO_2_]^-^, 111.0075[M-H-H_2_O-COOH-CO]^-^ | citric acid | all |
| 9 | 1.31 | C_16_H_24_O_9_ | 359.1346 | 1.205 | 197.0813[M-H-Glc]^-^, 179.0705[M-H-Glc-H_2_O]^-^ | 1-O-*β*-D-glucopyranosyl-  paeonisuffrone | PR |
| 10 | 1.68 | C_19_H_26_O_15_ | 493.1201 | 2.684 | 331.0661[M-H-Glc]^-^, 313.0564[M-H-Glc-H_2_O]^-^,  169.0133[M-H-C_12_H_20_O_10_]^-^, 125.0232[M-H-C_12_H_20_O_10_-CO_2_]^-^ | 6'-O-galloylsucrose | PR |
| 11 | 1.85 | C_19_H_26_O_15_ | 493.1198 | 2.056 | 331.0668[M-H-Glc]^-^, 313.0565[M-H-Glc-H_2_O]^-^,  169.0133[M-H-C_12_H_20_O_10_]^-^, 125.0232[M-H-C_12_H_20_O_10_-CO_2_]^-^ | 6-O-galloylsucrose | PR |
| 12* | 1.95 | C_7_H_6_O_5_ | 169.0132 | -2.947 | 125.0232[M-H-CO_2_]^-^ | gallic acid | PR |
| 13 | 2.24 | C_9_H_14_O_3_ | 169.0860 | 0.350 | 151.0750[M-H-H_2_O]^-^, 125.0231[M-H-CO_2_]^-^, 123.0803[M-H-CO-H_2_O]^-^ | isoboonein | GM |
| 14 | 2.55 | C_16_H_22_O_10_ | 373.1139 | 1.121 | 211.0605[M-H-Glc]^-^, 167.0703[M-H-Glc-CO_2_]^-^,  149.0597[M-H-Glc-CO_2_-H_2_O]^-^, 123.0439[M-H-Glc-CO_2_-H_2_O-C_2_H_2_]^-^ | geniposidic acid | GM, PS |
| 15 | 3.45 | C_7_H_6_O_4_ | 153.0182 | -4.010 | 109.0282[M-H-CO_2_]^-^, 91.0175[M-H-CO_2_-H_2_O]^-^ | protocatechuic acid | SG |
| 17 | 3.56 | C_16_H_22_O_10_ | 373.1138 | 0.960 | 211.0603[M-H-Glc]^-^, 167.0703[M-H-Glc-CO_2_]^-^, 139.0751[M-H-Glc-CO_2_-CO]^-^ | secologanic acid | GM |
| 19* | 3.79 | C_16_H_24_O_10_ | 375.1289 | 0.978 | 213.0762[M-H-Glc]^-^, 169.0860[M-H-Glc-CO_2_]^-^, 151.0755[M-H-Glc-CO_2_-H_2_O]^-^ | loganic acid | GM |
| 20 | 3.97 | C_16_H_18_O_9_ | 353.0876 | 2.383 | 191.0553[M-H-caffeoyl]^-^, 179.0430[M-H-C_7_H_10_O_5_]^-^, 135.0440[M-H-C_8_H_10_O_7_]^-^ | cryptochlorogenic acid isomer | PC |
| 22 | 4.12 | C_7_H_6_O_3_ | 137.0231 | -1.245 | 119.0124[M-H-H_2_O]^-^, 108.0204[M-H-CHO]^-^, 93.0333[M-H-CO_2_]^-^, 81.0332[M-H-2CO]^-^ | protocatechualdehyde | CTR, SG |
| 24* | 4.37 | C_16_H_22_O_10_ | 419.1189  [M+COOH]^-^ | 1.259 | 211.0607[M-H-Glc]^-^, 179.0552[C_6_H_11_O_6_]^-^, 149.0596[M-H-Glc-H_2_O- H_2_O-C_2_H_2_]^-^,  141.0182[M-H-Glc-H_2_O-C_4_H_4_]^-^, 119.0337[M-H-Glc-H_2_O- H_2_O-C_2_H_2_-CO]^-^ | swertiamain | GM |
| 26 | 4.49 | C_17_H_24_O_9_ | 417.1400  [M+COOH]^-^ | 1.947 | 209.0812[M-H-C_6_H_10_O_5_]^-^, 194.0577[M-H-C_6_H_10_O_5_-CH_3_]^-^,  179.0342[M-H-C_6_H_10_O_5_-2CH_3_]^-^, 161.0234[M-H-C_6_H_10_O_5_-2CH_3_-H_2_O]^-^ | syringin | PC |
| 27 | 4.52 | C_16_H_22_O_11_ | 389.1085 | 1.599 | 345.1187[M-H-CO_2_]^-^, 209.0446[M-H-C_6_H_10_O_5_-H_2_O]^-^, 165.0547[M-H-C_6_H_10_O_5_-H_2_O-CO_2_]^-^, 121.0646[M-H-C_6_H_10_O_5_-H_2_O-CO_2_-CO_2_]^-^ | secologanoside | GM |
| 31* | 5.08 | C_23_H_28_O_12_ | 495.1507 | 2.075 | 465.1407[M-H-CH_2_O]^-^, 137.0232[M-H-C_16_H_22_O_9_]^-^, 93.0332[M-H-C_16_H_22_O_9_-CO_2_]^-^ | oxypaeoniflorin | PR |
| 32* | 5.14 | C_16_H_20_O_9_ | 401.1082  [M+COOH]^-^ | 0.953 | 179.0552[C_6_H_11_O_6_]^-^, 149.0595[M-Glc-H_2_O-C_2_H_2_]^-^, 119.0338[M-Glc-H_2_O-C_2_H_2_-CO]^-^ | gentiopicroside | GM |
| 34 | 5.33 | C_16_H_22_O_9_ | 403.1240  [M+COOH]^-^ | 1.171 | 357.1197[M-H]^-^, 195.0654[M-H-Glc]^-^, 179.0553[C_6_H_11_O_6_]^-^,  125.0232[M-H-Glc-H_2_O-C_4_H_4_]^-^ | sweroside | GM |
| 35 | 5.42 | C_7_H_6_O_3_ | 137.0232 | -0.734 | 93.0333[M-H-CO_2_]^-^ | 4-hydroxybenzoic acid | PR |
| 36 | 5.45 | C_17_H_20_O_9_ | 367.1023 | -0.187 | 193.0498[M-H-C_7_H_10_O_5_]^-^, 134.0361[M-H-CH_3_-C_8_H_10_O_7_]^-^ | 3‑O‑feruloylquinic acid | PC |
| 37* | 5.47 | C_16_H_18_O_9_ | 353.0875 | 2.218 | 191.0553[M-H-caffeoyl]^-^, 179.0340[M-H-C_7_H_10_O_5_]^-^, 173.0445[M-H-C_9_H_8_O_4_]^-^, 135.0440[M-H-C_7_H_10_O_5_-CO_2_]^-^, 93.0332[M-H-2H_2_O-CO_2_]^-^ | cryptochlorogenic acid | PC |
| 40* | 5.53 | C_15_H_14_O_6_ | 289.0715 | 2.890 | 245.0815[M-H-CO_2_]^-^, 203.0705[M-H-C_3_H_2_O_3_]^-^, 123.0439[M-H-C_8_H_6_O_4_]^-^, 109.0282[M-H-C_9_H_8_O_4_]^-^ | catechin | SG |
| 44* | 5.82 | C_16_H_18_O_9_ | 353.0872 | 1.534 | 191.0553 [M-H-caffeoyl]^-^, | chlorogenic acid | PC |
| 45* | 5.88 | C_23_H_28_O_11_ | 525.1605 | 0.481 | 357.1181[M-H-C_7_H_6_O_2_]^-^, 283.0829[M-H-C_10_H_12_O_4_]^-^, 121.0283[M-H-C_16_H_22_O_9_]^-^ | alibiflorin | PR |
| 46 | 5.97 | C_16_H_18_O_9_ | 353.0877 | 2.836 | 191.0553[M-H-caffeoyl]^-^ | 1-caffeoylquinic acid | PC |
| 50* | 6.47 | C_15_H_14_O_6_ | 289.0714 | 2.579 | 245.0816[M-H-CO_2_]^-^, 203.0702[M-H-C_3_H_2_O_3_]^-^, 123.0439[M-H-C_8_H_6_O_4_]^-^, 109.0282[M-H-C_9_H_8_O_4_]^-^ | epicatechin | SG |
| 51* | 6.54 | C_23_H_28_O_11_ | 525.1603  [M+COOH]^-^ | 0.138 | 449.1450[M-H-CH_2_O]^-^, 327.1081[M-H-C_7_H_6_O_2_-CH_2_O]^-^,  165.0547[M-H-C_7_H_6_O_2_-CH_2_O-Glc]^-^, 121.0282[M-H-C_16_H_22_O_9_]^-^ | paeoniflorin | PR |
| 56 | 7.12 | C_17_H_20_O_9_ | 367.1021 | -0.677 | 193.0499[M-H-C_7_H_10_O_5_]^-^, 173.0446[M-H-C_10_H_8_O_3_-H_2_O]^-^, 134.0361[M-H-CH_3_-C_8_H_10_O_7_]^-^ | 4‑O‑feruloylquinic acid | PC |
| 59 | 7.56 | C_16_H_18_O_8_ | 337.0927 | 2.629 | 191.0552[M-H-C_9_H_6_O_2_]^-^, 173.0445[M-H-C_9_H_6_O_2_-H_2_O]^-^,  163.0389[M-H-C_7_H_10_O_5_]^-^, 119.0489[M-H-C_7_H_10_O_5_-CO_2_]^-^ | 4-p-coumaroylquinic acid | PC |
| 61 | 7.68 | C_17_H_20_O_9_ | 367.1022 | -0.350 | 191.0552[M-H-C_10_H_8_O_3_]^-^, 173.0446[M-H-C_10_H_8_O_3_-H_2_O]^-^,  134.0361[M-H-CH_3_-C_8_H_10_O_7_]^-^, 111.0438[M-H-C_10_H_9_O_4_-CO_2_-OH]^-^ | 5‑O‑feruloylquinic acid | PC |
| 64 | 7.88 | C_16_H_16_O_8_ | 335.0768 | 1.988 | 179.0340[M-H-C_7_H_8_O_4_]^-^, 173.0446[M-H-C_9_H_6_O_3_]^-^, 161.0233[M-H-C_7_H_8_O_4_-H_2_O]^-^,  135.0439[M-H-C_7_H_8_O_4_-CO_2_]^-^ | 5-O-caffeoylshikimic acid | SG |
| 72 | 8.38 | C_26_H_34_O_11_ | 521.2021 | 0.675 | 359.1503[M-H-C_6_H_10_O_5_]^-^, 329.1392[M-H-C_7_H_12_O_6_]^-^,  175.0755[M-H-C_15_H_22_O_9_]^-^, 160.0519[M-H-C_15_H_22_O_9_-CH_3_]^-^ | A_2_ | PC |
| 73 | 8.49 | C_30_H_26_O_11_ | 561.1396 | 0.913 | 543.1301[M-H-H_2_O]^-^, 435.1088[M-H-H_2_O-C_6_H_4_O_2_]^-^, 289.0716[M-H-C_15_H_12_O_5_]^-^, 273.0764[M-H-C_15_H_12_O_6_]^-^, 271.0610[M-H-C_15_H_12_O_5_-H_2_O]^-^, 245.0814[M-H-C_15_H_12_O_6_-CO]^-^, 125.0231[M-H-C_15_H_12_O_5_-H_2_O-C_9_H_6_O_3_]^-^ | catechin-(4alpha->8)-  epiafzelechin or its isomer | SG |
| 78 | 8.95 | C_9_H_8_O_3_ | 163.0389 | -0.249 | 119.0489[M-H-CO_2_]^-^ | p-hydroxy-cinnamic acid | PR, PC |
| 82 | 9.13 | C_21_H_22_O_11_ | 449.1084 | 1.252 | 269.0453[M-H-C_6_H_12_O_6_]^-^,178.9976[M-H-C_6_H_12_O_6_-C_7_H_6_]^-^, 151.0026[M-H-C_6_H_12_O_6_-C_7_H_6_-CO]^-^ | astilbin isomer | SG |
| 83 | 9.14 | C_30_H_32_O_15_ | 631.1660 | 0.433 | 465.1214[M-H-C_7_H_5_O-H_2_O-CO_2_]^-^, 313.0568[M-H-C_17_H_17_O_5_-H_2_O]^-^,  169.0136[M-H-C_23_H_27_O_10_]^-^, 121.0285[M-H-C_23_H_27_O_13_]^-^ | galloylpaeoniflorin | PR |
| 84 | 9.14 | C_29_H_36_O_15_ | 623.1976 | 0.840 | 461.1657[M-H-C_9_H_6_O_3_]^-^, 179.0341[M-H-C_20_H_28_O_11_]^-^, 161.0234[M-H-C_20_H_28_O_11_-H_2_O]^-^,  133.0283[M-H-C_20_H_28_O_11_-H_2_O-CO]^-^ | acteoside | PS |
| 87 | 9.40 | C_22_H_26_O_8_ | 417.1550 | 1.524 | 402.1309[M-H-CH_3_]^-^, 387.1075[M-H-2CH_3_]^-^, 181.0496[M-H-C_13_H_13_O_4_]^-^, 166.0261[M-H-C_13_H_13_O_4_-CH_3_]^-^,151.0025[M-H-C_13_H_13_O_4_-2CH_3_]^-^ | syringaresinol | GM, SG |
| 90 | 9.71 | C_24_H_20_O_9_ | 451.1029 | 1.134 | 341.0664[M-H-C_6_H_6_O_2_]^-^, 217.0135[M-H-C_13_H_14_O_4_]^-^, 189.0185[C_10_H_5_O_4_]^-^, 109.0282[C_6_H_5_O_2_]^-^ | cinchonain Ia or its isomer | SG |
| 92 | 9.99 | C_9_H_16_O_4_ | 187.0966 | 0.612 | 169.0860[M-H-H_2_O]^-^, 143.1066[M-H-CO_2_]^-^, 125.0959[M-H-H_2_O-CO_2_]^-^, 97.0645[M-H-CO_2_-C_2_H_4_]^-^ | azelaic acid | GM |
| 93 | 10.04 | C_15_H_12_O_7_ | 303.0508 | 2.808 | 285.0402[M-H-H_2_O]^-^, 177.0183[M-H-H_2_O]^-^, 151.0026[M-H-C_7_H_4_O_4_]^-^, 125.0231[M-H-C_9_H_6_O_4_]^-^ | toxifolin | SG |
| 94* | 10.11 | C_29_H_44_O_8_ | 565.3016  [M+COOH]^-^ | 1.497 | 519.2958[M-H]^-^, 501.2894[M-H-H_2_O]^-^, 319.1905[M-H-C_10_H_16_O_4_]^-^, 301.1810[M-H-C_10_H_16_O_4_-H_2_O]^-^, 199.0967[C_10_H_15_O_4_]^-^ | cyasterone | CTR |
| 95 | 10.12 | C_21_H_22_O_11_ | 449.1084 | 1.252 | 303.0508[M-H-C_6_H_10_O_4_]^-^, 285.0403[M-H-C_6_H_10_O_4_-H_2_O]^-^, 151.0026[M-H-C_6_H_10_O_4_-C_8_H_8_O_3_]^-^ | astilbin isomer | SG |
| 96 | 10.15 | C_29_H_36_O_15_ | 623.1975 | 0.744 | 461.1662[M-H-C_9_H_6_O_3_]^-^, 179.0340[M-H-C_20_H_28_O_11_]^-^, 161.0233[M-H-C_20_H_28_O_11_-H_2_O]^-^,  133.0282[M-H-C_20_H_28_O_11_-H_2_O-CO]^-^ | isoacteoside | PS |
| 98 | 10.36 | C_21_H_22_O_11_ | 449.1082 | 0.695 | 303.0508[M-H-C_6_H_10_O_4_]^-^, 285.0403[M-H-C_6_H_10_O_4_-H_2_O]^-^,  151.0026[M-H-C_6_H_10_O_4_-C_8_H_8_O_3_]^-^ | neoastilbin | SG |
| 99 | 10.38 | C_30_H_32_O_15_ | 631.1662 | 0.718 | 313.0562[M-H-C_17_H_17_O_5_-H_2_O]^-^, 169.0137[M-H-C_23_H_27_O_10_]^-^, 121.0286[M-H-C_23_H_27_O_13_]^-^ | galloylpaeoniflorin isomer | PR |
| 100 | 10.49 | C_24_H_20_O_9_ | 451.1030 | 1.488 | 341.0663[M-H-C_6_H_6_O_2_]^-^, 217.0135[M-H-C_13_H_14_O_4_]^-^, 189.0184[C_10_H_5_O_4_]^-^, 109.0282[C_6_H_5_O_2_]^-^ | cinchonain Ia or its isomer | SG |
| 101* | 10.57 | C_7_H_6_O_3_ | 137.0231 | -1.902 | 93.0332[M-H-CO_2_]^-^ | salicylic acid | PR |
| 103* | 10.63 | C_21_H_22_O_11_ | 449.1082 | 0.695 | 303.0508[M-H-C_6_H_10_O_4_]^-^, 285.0403[M-H-C_6_H_10_O_4_-H_2_O]^-^,  151.0026[M-H-C_6_H_10_O_4_-C_8_H_8_O_3_]^-^ | astilbin | SG |
| 104 | 10.74 | C_24_H_20_O_9_ | 451.1031 | 1.555 | 341.0663[M-H-C_6_H_6_O_2_]^-^, 217.0135[M-H-C_13_H_14_O_4_]^-^, 189.0184[C_10_H_5_O_4_]^-^, 109.0282[C_6_H_5_O_2_]^-^ | cinchonain Ia or its isomer | SG |
| 107 | 11.61 | C_21_H_20_O_11_ | 447.0934 | 1.101 | 301.0347[M-H-C_6_H_10_O_4_]^-^, 300.0273[M-H-C_6_H_11_O_4_]^-^, 271.0247[M-H-C_7_H_12_O_5_]^-^, 255.0296[M-H-C_7_H_12_O_6_]^-^, 178.9977[M-H-C_13_H_16_O_6_]^-^, 151.0026[M-H-C_14_H_16_O_7_]^-^ | quercitrin | SG |
| 109 | 11.75 | C_21_H_22_O_10_ | 433.1133 | 0.824 | 287.0556[M-H-C_6_H_10_O_4_]^-^, 269.0454[M-H-C_6_H_12_O_5_]^-^, 259.0608[M-H-C_7_H_10_O_5_]^-^, 178.9977[M-H-C_13_H_18_O_5_]^-^, 152.0104[M-H-C_14_H_17_O_6_]^-^ | neoengeletin | SG |
| 110 | 11.94 | C_24_H_20_O_9_ | 451.1028 | 1.067 | 341.0664[M-H-C_6_H_6_O_2_]^-^, 217.0135[M-H-C_13_H_14_O_4_]^-^, 189.0185[C_10_H_5_O_4_]^-^, 109.0282[C_6_H_5_O_2_]^-^ | cinchonain Ia or its isomer | SG |
| 111* | 12.04 | C_21_H_22_O_10_ | 433.1132 | 0.616 | 287.0560[M-H-C_6_H_10_O_4_]^-^, 269.0457[M-H-C_6_H_12_O_5_]^-^, 259.0606[M-H-C_7_H_10_O_5_]^-^, 178.9977[M-H-C_13_H_18_O_5_]^-^, 152.0105[M-H-C_14_H_17_O_6_]^-^ | engeletin | SG |
| 112 | 12.31 | C_15_H_12_O_6_ | 287.0559 | 3.224 | 259.0609[M-H-CO]^-^, 151.0021[M-H-C_8_H_6_O_2_]^-^,  125.0231[M-H-C_9_H_6_O_3_]^-^, 107.0124[M-H-C_9_H_8_O_4_]^-^ | eriodictyol isomer | PR, SG |
| 114 | 13.97 | C_15_H_12_O_6_ | 287.0558 | 2.910 | 151.0026[M-H-C_8_H_6_O_2_]^-^, 135.0439[M-H-C_7_H_4_O_4_]^-^, 125.0229[M-H-C_9_H_6_O_3_]^-^, 107.0125[M-H-C_9_H_8_O_4_]^-^ | eriodictyol | PR, PS |
| 115 | 14.09 | C_26_H_30_O_9_ | 485.1815 | 1.816 | 345.1704[M-H-C_6_H_4_O_4_]^-^, 317.1768[M-H-C_7_H_4_O_5_]^-^, 205.0862[M-H-C_14_H_16_O_6_]^-^, 161.0962[M-H-C_15_H_16_O_8_]^-^, 129.0544[M-H-C_20_H_20_O_6_]^-^ | rutaevin isomer | PC |
| 116 | 14.49 | C_30_H_32_O_12_ | 629.1868  [M+COOH]^-^ | 0.553 | 553.1716[M-H-CH_2_O]^-^, 431.1352[M-H-C_8_H_8_O_3_]^-^, 121.0282[C_7_H_5_O_2_]^-^ | benzoylpaeoniflorin | PR |
| 118 | 14.73 | C_30_H_32_O_12_ | 629.1869  [M+COOH]^-^ | 0.648 | 553.1726[M-H-CH_2_O]^-^, 431.1359[M-H-C_8_H_8_O_3_]^-^, 121.0282[C_7_H_5_O_2_]^-^ | isobenzoylpaeoniflorin | PR |
| 119 | 15.12 | C_40_H_44_O_23_ | 891.2200 | 1.129 | 755.2039[M-H-C_7_H_4_O_3_]^-^, 593.1512[M-H-C_13_H_14_O_8_]^-^, 315.0721[C_13_H_15_O_9_]^-^, 153.0183[C_7_H_5_O_4_]^-^, 141.0182[C_6_H_5_O_4_]^-^, 109.0282[C_6_H_5_O_2_]^-^ | macrophylloside B | GM |
| 120 | 15.62 | C_40_H_44_O_22_ | 875.2252 | 1.338 | 739.2106[M-H-C_7_H_4_O_3_]^-^, 577.1559[M-H-C_13_H_14_O_8_]^-^, 315.0721[C_13_H_15_O_9_]^-^, 153.0182[C_7_H_5_O_4_]^-^, 125.0232[C_6_H_5_O_3_]^-^, 109.0282[C_6_H_5_O_2_]^-^ | macrophylloside A | GM |
| 121* | 15.64 | C_15_H_10_O_6_ | 285.0403 | 3.107 | 151.0025[M-H-C_8_H_6_O_2_]^-^, 133.0283[M-H-C_7_H_4_O_4_]^-^, 107.0126[M-H-C_9_H_6_O_4_]^-^ | luteolin | CTR, PR  SG, PS |
| 123 | 15.97 | C_26_H_30_O_9_ | 485.1816 | 2.125 | 423.1801[M-H-CH_2_O_3_]^-^, 383.1498[M-H-C_4_H_6_O_3_]^-^, 259.1334[M-H-C_10_H_10_O_6_]^-^, 166.0259[M-H-C_18_H_23_O_5_]^-^, 123.0439[M-H-C_19_H_22_O_7_]^-^ | rutaevin | PC |
| 124* | 15.99 | C_15_H_12_O_5_ | 271.0609 | 3.099 | 177.0185[M-H-C_6_H_6_O]^-^, 151.0027[M-H-C_8_H_8_O]^-^, 119.0490[M-H-C_7_H_4_O_4_]^-^, 107.0126[M-H-C_9_H_8_O_3_]^-^ | naringenin | SG |
| 125 | 16.06 | C_48_H_76_O_18_ | 939.4961 | 0.798 | 777.4437[M-H-C_6_H_10_O_5_]^-^, 715.4435[M-H-C_6_H_10_O_5_-CH_2_O_3_]^-^, 569.3849[M-H-  C_13_H_22_O_12_]^-^, 551.3735[M-H-C_13_H_22_O_12_-H_2_O]^-^, 455.3531[M-H-C_18_H_28_O_15_]^-^ | momordin IIa | CTR |
| 126 | 16.21 | C_42_H_66_O_14_ | 793.4379 | 0.553 | 631.3862[M-H-C_6_H_10_O_5_]^-^, 569.3846[M-H-C_7_H_12_O_8_]^-^, 455.3534[M-H-C_12_H_18_O_11_]^-^ | chikusetsu saponin IVa | CTR |
| 127 | 16.28 | C_15_H_12_O_5_ | 271.0610 | 3.468 | 177.0185[M-H-C_6_H_6_O]^-^, 151.0027[M-H-C_8_H_8_O]^-^, 119.0490[M-H-C_7_H_4_O_4_]^-^, 107.0126[M-H-C_9_H_8_O_3_]^-^ | naringenin isomer | SG |
| 130 | 16.64 | C_15_H_10_O_5_ | 269.0454 | 3.569 | 225.0553[M-H-CO]^-^, 151.0026[M-H-C_8_H_6_O]^-^, 117.0333[M-H-C_7_H_4_O_4_]^-^, 107.0125[M-H-C_9_H_6_O_3_]^-^ | apigenin | PR, PS |
| 131 | 16.73 | C_30_H_46_O_6_ | 501.3221 | 1.964 | 455.3157[M-H-CH_2_O_2_]^-^, 405.2804[M-H-C_2_H_8_O_4_]^-^, 389.2853[M-H-C_2_H_8_O_5_]^-^, 373.2526[M-H-C_3_H_12_O_5_]^-^ | A_3_ | GM |
| 136 | 17.22 | C_48_H_76_O_18_ | 939.4960 | 0.671 | 793.4410[M-H-C_6_H_10_O_4_]^-^, 551.3769[M-H-C_13_H_22_O_12_-H_2_O]^-^, 455.3534[M-H-C_18_H_28_O_15_]^-^ | momordin Iia isomer | CTR |
| 137 | 17.22 | C_42_H_66_O_14_ | 793.4379 | 0.628 | 567.3701[M-H-C_7_H_14_O_8_]^-^, 471.3530[M-H-C_19_H_14_O_5_]^-^, 439.3220[M-H-C_13_H_22_O_11_]^-^ | chikusetsu saponin IVa isomer | CTR |

Note: *t*_R_: retention time; *: compound identified by compared with a reference standard; Glc: *β*-D-glucose; GM: *Gentianae Macrophyllae* Radix; PC: *Phellodendri Chinensis* Cortex;

CR: *Corydalis* Rhizoma; PR: *Paeoniae* Radix Rubra; CTR: *Cyathulae* Radix; AR: *Alismatis* Rhizoma; PS: *Plantaginis* Semen; SG: *Smilacis Glabrae* Rhizoma.

A_2_: (+/-) 8-( 4-hydroxy-3-methoxyphenyl) -6,7-bis(hydroxymethyl) -3-methoxy-5,6,7,8-tetrahydro-2-naphthalenyl-*β*-D-glucopyranoside.

A_3_: 1,2,3,24-tetrahydroxyursa-12,20(30)-dien-28-oic acid

**Supplementary Table 3.** Chemical structures of the absorbed prototype component.

| Peak No. | Identified Compound | Structure |
| --- | --- | --- |
| 3 | adenosine |  |
| 6 | malic acid |  |
| 8 | citric acid |  |
| 14 | geniposidic acid |  |
| 18 | lotusine |  |
| 19 | loganic acid |  |
| 21 | A_1_ |  |
| 22 | protocatechualdehyde |  |
| 25 | phellodendrine |  |
| 26 | syringin |  |
| 27 | secologanoside |  |
| 28 | 6'-O-*β*-D-glucosyl-gentiopicroside |  |
| 29 | N-methylhigenamine-7-O-glucopyranoside |  |
| 30 | magnoflorine |  |
| 31 | oxypaeoniflorin |  |
| 32 | gentiopicrin |  |
| 39 | oblongine |  |
| 41 | plantagoguanidinic acid |  |
| 42 | menisperine |  |
| 44 | chlorogenic acid |  |
| 45 | alibiflorin |  |
| 49 | tembetarine |  |
| 51 | paeoniflorin |  |
| 52 | yuanhunine |  |
| 53 | tetrahydrocolumbamine |  |
| 55 | protopine |  |
| 57 | berberastine |  |
| 58 | demethyleneberberine |  |
| 59 | 4-p-Coumaroylquinic acid |  |
| 61 | 3‑O‑feruloylquinic acid |  |
| 62 | allocryptopine |  |
| 64 | 5-O-caffeoylshikimic acid |  |
| 65 | N-methyltetrahy-dropalmatine |  |
| 66 | tetrahydropalmatine |  |
| 67 | glaucine |  |
| 68 | coptisine |  |
| 71 | columbamine |  |
| 74 | jatrorrhizine |  |
| 75 | tetrahydroberberine |  |
| 76 | thalifendine |  |
| 79 | corydaline |  |
| 80 | corysamine |  |
| 85 | berberrubine |  |
| 86 | 13-methyl-dehydro-corydalmine |  |
| 88 | berberine |  |
| 89 | palmatine |  |
| 92 | azelaic acid |  |
| 94 | cyasterone |  |
| 97 | dehydrocorydaline |  |
| 98 | neoastilbin |  |
| 101 | salicylic acid |  |
| 103 | astilbin |  |
| 109 | neoengeletin |  |
| 111 | engeletin |  |
| 116 | benzoylpaeoniflorin |  |
| 117 | γ-fagarine |  |
| 118 | isobenzoylpaeoniflorin |  |
| 125 | Momordin Iia |  |
| 130 | apigenin |  |

Note: A_1_: 3,4-Dihydro-1-[(4-hydroxyphenyl)methyl]-7-methoxy-2-methyl-6-isoquinolinol.
